# Multi-ancestry meta-analysis and fine-mapping in Alzheimer’s disease

**DOI:** 10.1038/s41380-023-02089-w

**Published:** 2023-05-18

**Authors:** Julie Lake, Caroline Warly Solsberg, Jonggeol Jeffrey Kim, Juliana Acosta-Uribe, Mary B. Makarious, Zizheng Li, Kristin Levine, Peter Heutink, Chelsea X. Alvarado, Dan Vitale, Sarang Kang, Jungsoo Gim, Kun Ho Lee, Stefanie D. Pina-Escudero, Luigi Ferrucci, Andrew B. Singleton, Cornelis Blauwendraat, Mike A. Nalls, Jennifer S. Yokoyama, Hampton L. Leonard

**Affiliations:** 1 Laboratory of Neurogenetics, National Institute on Aging, National Institutes of Health, Bethesda, MD USA; 2 Pharmaceutical Sciences and Pharmacogenomics, University of California, San Francisco, San Francisco, CA USA; 3 Department of Neurology and Weill Institute for Neurosciences, University of California, San Francisco, San Francisco, CA USA; 4 Memory and Aging Center, University of California, San Francisco, San Francisco, CA USA; 5 Preventive Neurology Unit, Centre for Prevention Diagnosis and Detection, Wolfson Institute of Population Health, Queen Mary University of London, London, UK; 6 Neuroscience Research Institute and the department of Molecular, Cellular and Developmental Biology, University of California, Santa Barbara, Santa Barbara, CA USA; 7 Neuroscience Group of Antioquia, University of Antioquia, Medellín, Colombia; 8 Department of Clinical and Movement Neurosciences, UCL Queen Square Institute of Neurology, London, UK; 9 UCL Movement Disorders Centre, University College London, London, UK; 10 Data Tecnica International LLC, Washington, DC USA; 11 Center for Alzheimer’s and Related Dementias, National Institutes of Health, Bethesda, MD USA; 12 Alector, Inc. 131 Oyster Point Blvd, Suite 600, South San Francisco, CA 94080 USA; 13 Gwangju Alzheimer’s disease and Related Dementia Cohort Research Center, Chosun University, Gwangju, 61452 Korea; 14 BK FOUR Department of Integrative Biological Sciences, Chosun University, Gwangju, 61452 Korea; 15 Department of Biomedical Science, Chosun University, Gwangju, 61452 Korea; 16 Korea Brain Research Institute, Daegu, 41062 Korea; 17 Longitudinal Studies Section, National Institute on Aging, National Institutes of Health, Baltimore, MD USA; 18 Integrative Neurogenomics Unit, Laboratory of Neurogenetics, National Institute on Aging, National Institutes of Health, Bethesda, MD USA; 19 Department of Radiology and Biomedical Imaging, University of California, San Francisco, San Francisco, CA USA; 20 German Center for Neurodegenerative Diseases (DZNE), Tübingen, Germany

**Keywords:** Neuroscience, Genetics, Diseases

## Abstract

Genome-wide association studies (GWAS) of Alzheimer’s disease are predominantly carried out in European ancestry individuals despite the known variation in genetic architecture and disease prevalence across global populations. We leveraged published GWAS summary statistics from European, East Asian, and African American populations, and an additional GWAS from a Caribbean Hispanic population using previously reported genotype data to perform the largest multi-ancestry GWAS meta-analysis of Alzheimer’s disease and related dementias to date. This method allowed us to identify two independent novel disease-associated loci on chromosome 3. We also leveraged diverse haplotype structures to fine-map nine loci with a posterior probability >0.8 and globally assessed the heterogeneity of known risk factors across populations. Additionally, we compared the generalizability of multi-ancestry- and single-ancestry-derived polygenic risk scores in a three-way admixed Colombian population. Our findings highlight the importance of multi-ancestry representation in uncovering and understanding putative factors that contribute to risk of Alzheimer’s disease and related dementias.

## Introduction

Alzheimer’s disease (AD) is a complex genetic disorder with a range of deleterious variants across multiple genes attributed to both early and late-onset forms of sporadic AD [1]. The strongest genetic risk factor for late-onset AD is *APO*E-e4, yet it has been estimated that there may be anywhere from 100 to 11,000 variants that also contribute to risk of late-onset AD [2, 3]. Large-scale genome-wide association studies (GWAS) in European ancestry populations have identified over 75 loci that are associated with AD and related dementias (ADD) [4]. However, genetic research in ADD that focuses solely on European populations limits additional discoveries afforded by studying diverse cohorts. Including non-European populations in genetic research provides new opportunities to uncover ancestry-specific risk variants and loci, increase statistical discovery power, improve fine-mapping resolution to identify putative causal variants, and identify loci with heterogeneous effects across ancestry groups [5–7].

Implementing existing ancestry-aware or heterogeneity penalizing meta-regression approaches have proven powerful at deconvoluting the genetic architecture of other phenotypes across populations [8–18]. We leveraged such techniques, layering existing diverse data on top of more extensive European-derived data, to facilitate discovery of novel ADD risk loci. Here we report the results of our multi-ancestry genome-wide meta-analysis of the largest publicly available ADD GWAS from individuals of European, East Asian, and African American ancestry, and an additional GWAS of Caribbean Hispanic individuals using previously reported genotype data since those summary statistics were not available. Using a meta-regression approach implemented in MR-MEGA, we demonstrate improved fine-mapping at several known ADD loci and estimate the extent to which heterogeneity at these loci is attributable to genetic ancestry. This study highlights the utility of multi-ancestry analyses to further our understanding of disease biology and reduce health disparities in research by nominating novel loci and characterizing genetic differences across populations.

## Results

### Data included in this study

**Fig. 1:**
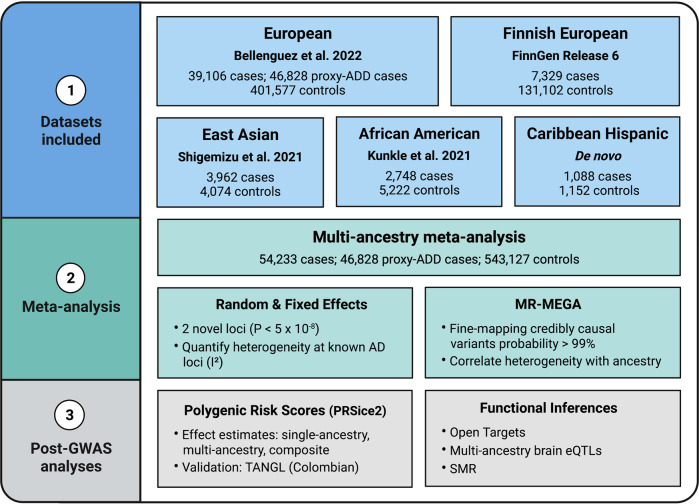
Study Design. Outline of multi-ancestry meta-analysis procedure and downstream analysis.

Our multi-ancestry meta-analysis included a total of 54,233 AD cases, 46,828 proxy AD and related dementia (proxy-ADD) cases, and 543,127 controls (Fig. 1 and Table S1). Detailed information about the existing GWAS summary statistics used in this report are described elsewhere [4, 6, 19]. In brief, the most recent publicly available ADD GWAS includes 39,106 clinically diagnosed AD cases, 46,828 proxy-ADD cases (defined as having a parent with AD/dementia) and 401,577 controls of European ancestry [4]. FinnGen data from Release 6 includes 7329 AD cases and 131,102 controls free of any neurological disorder. We also included the largest publicly available AD GWAS of African American (2748 cases and 5222 controls) [6] and East Asian (3962 cases and 4074 controls) [19] populations and an additional GWAS including 1095 cases and 1179 controls of Caribbean Hispanic ancestry. Select SNPs from the Gwangju Alzheimer’s & Related Dementias (GARD) East Asian cohort (1119 cases and 1172 controls) were used to assess East Asian risk at our novel loci post-hoc since these SNPs were not present in the discovery East Asian dataset from Shigemizu et al. used in our meta-GWAS [20]. In this study, we considered significant variants as passing the standard *p* value threshold of 5 × 10^8^, consistent with most GWAS meta-analyses and used previously in other multi-ancestry studies [21–23]. Our analysis included only variants that passed quality control and with a minor allele frequency >1% in a minimum of three datasets to accurately quantify heterogeneity, effectively reducing the number of potential haplotypes and tests.

### Meta-GWAS

Association summary statistics from all five datasets, representing four super populations, were aggregated via fixed and random effects models implemented in PLINK v1.9 [24] and a multi-ancestry meta-regression implemented in MR-MEGA [25] (Table S1). A fixed effect analysis was conducted in conjunction with random effects in PLINK as it is the standard choice for many GWAS meta-analyses. Since the fixed effect analysis did not identify any additional novel loci, we focused on the random effects and MR-MEGA results as these methods are generally more appropriate for multi-ancestry studies. In particular, MR-MEGA was specifically designed for multi-ancestry meta-analyses and random effects models penalize heterogeneity in their construction of effect estimates, allowing these estimates to be more generalizable across global populations. We did not observe any genomic inflation in these analyses after excluding rare variants (MAF < 1% per study) and correcting for case-control imbalance (Table S1 and Fig. S1). Chromosome 19 was also excluded from genomic inflation estimates to avoid bias from the *APOE* region. Association results from the random effects and MR-MEGA meta-analyses were moderately concordant for SNPs without heterogeneity (*I*^2^ = 0, *R*^2^ = 0.6). Our study also demonstrated that MR-MEGA is advantageous for SNPs with heterogeneous allelic effects (Fig. S2). Results from all meta-analyses (MR-MEGA, random effects, and fixed effect) can be found in Table S2a–c.

A total of 68 loci reached genome-wide significance (*P* < 5 × 10^−8^) in the fixed effect, random effects or MR-MEGA meta-analyses. While most of these loci overlapped all analyses, 1 was only significant using MR-MEGA (*JAZF1*), 1 using random effects (*KANSL1*), and 5 using the fixed effect model (*ADAMTS1*, *MAF*, *PLEKHA1*, *TSPOAP1*, and *UMAD1*) (Table S2a–c). 66 of these loci overlapped previously established genomic regions associated with ADD (see “Defining associated loci” in the “Online Methods”). Our analysis may have been underpowered to detect the remaining loci without the replication summary statistics from Bellenguez et al. as only the discovery phase statistics from that study were available.

**Fig. 2:**
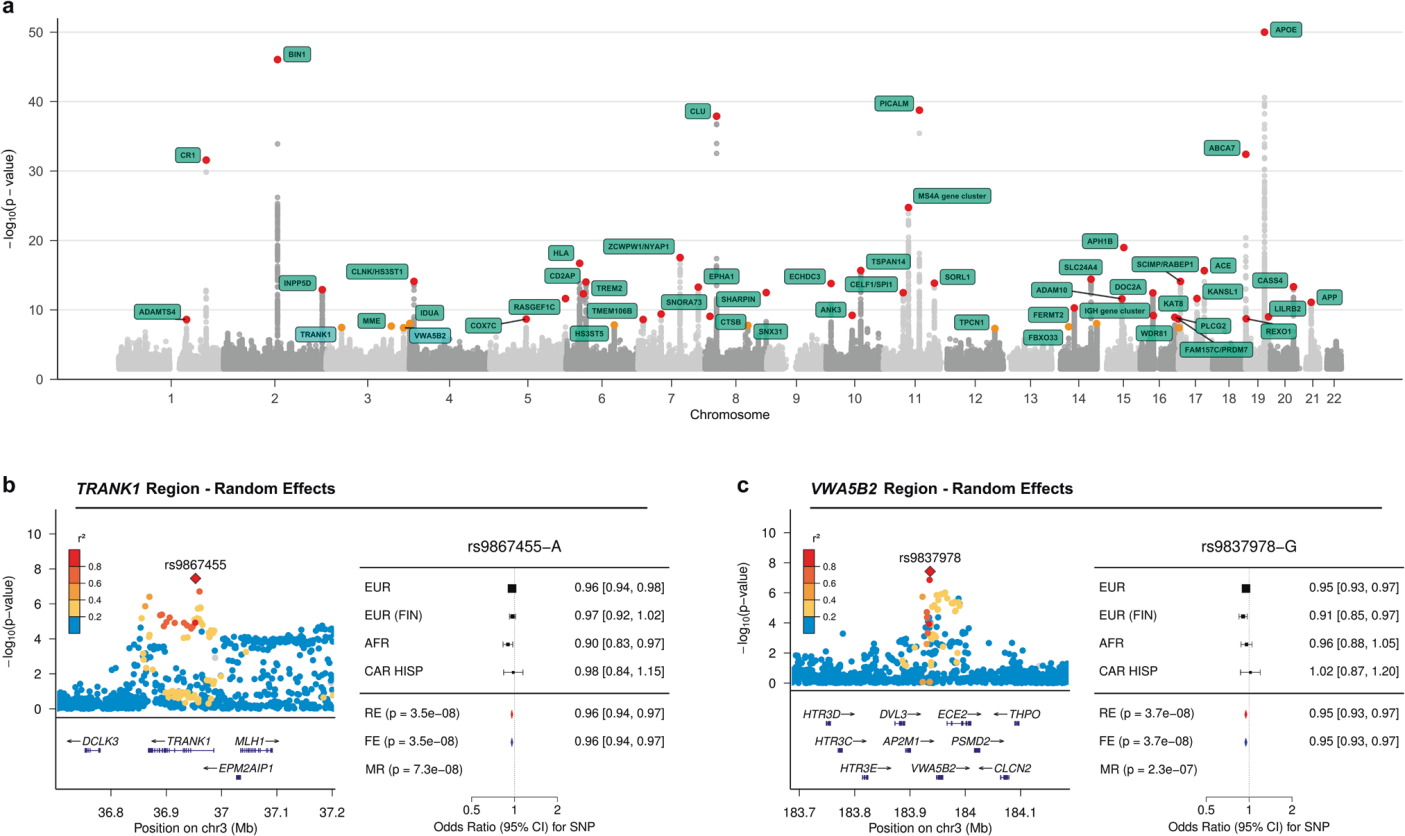
Summary of multi-ancestry meta-analysis. **a** The Manhattan plot for the random effects meta-analysis *P* values, truncated at −log10(P) < 50. An orange dot indicates that the lead SNP at a locus reached a *P* value < 5 × 10^-8^, while a red dot indicates a *P* value < 5 × 10^−9^. **b**, **c** The corresponding local association plots for the two loci of interest and forest plots summarizing the effect estimates per ancestry group for lead SNPs at the two loci of interest. Lead SNPs from both novel loci were absent in the East Asian dataset by Shigemizu et al. used for discovery. Abbreviations - MR MR-MEGA, RE Random effects, FE Fixed effect, EUR European, EUR (FIN) Finnish European, AFR African American, CAR HISP Caribbean Hispanic.

We additionally identified two independent ADD risk loci on chromosome 3 near *TRANK1* (rs9867455; *P*_RE_ = 3.49 × 10^−8^, *β*_RE_ = −0.0424, *I*^2^ = 0) and *VWA5B2* (rs9837978; *P*_RE_ = 3.75 × 10^−8^, *β*_RE_ = −0.0526, *I*^2^ = 0) that are outside of the maximal linkage disequilibrium (LD) boundary for any known AD risk loci (Fig. 2). These two loci were identified using both the fixed and random effects models. These loci also showed similar *P* values using MR-MEGA, but did not reach genome-wide significance using this method (Table 1). The association signals are primarily driven by the European-focused study by Bellenguez et al., where they were also sub-significant (P-values of 6.95 × 10^−7^ for *TRANK1* and 1.17 × 10^−6^ for *VWA5B2*, respectively). We did not identify any additional novel loci using MR-MEGA (Fig. S12a).

**Table 1:** Summary of novel loci.

Locus	SNP	Chromosome	Position	Effect allele	Reference allele	P, MR-MEGA	P, ancestry heterogeneity	P, random effects	beta, random effects	SE, random effects	I2	Mean effect allele frequency	Minimum effect allele frequency	Maximum effect allele frequency
*VWA5B2*	rs9837978	3	183936947	G	A	2.32E−07	5.96E−01	3.75E−08	−0.053	0.009	0.000	0.287	0.171	0.450
*TRANK1*	rs9867455	3	36953424	A	T	7.33E−08	1.18E−01	3.49E−08	−0.042	0.008	0.000	0.405	0.326	0.503

Since the lead SNPs for these potential novel loci were absent in the East Asian dataset from Shigemizu et al. used for initial discovery [19], we attempted to test these SNPs in an independent East Asian ancestry dataset [20]. Genome-wide data for this cohort was not available to include in the meta-analysis at the time of publication. We observed an association at *P* < 0.05 at *VWA5B2*-rs9837978 (*P* = 0.048, β = 0.204) in the GARD cohort (*n* = 2,291). The direction of effect was not consistent with the direction seen in the other populations included in the discovery GWAS, suggesting the effect of this locus may be heterogeneous across populations, but more extensive testing in East Asian populations must be performed for confirmation. We were unable to test the association at *TRANK1*-rs9867455 in the GARD replication cohort since this SNP was not included in their GWAS and an LD proxy SNP was not available.

### Gene prioritization for novel loci

Using public expression quantitative trait locus (eQTL) evidence from Open Targets [26] and multi-ancestry brain eQTL summary data [27], we assessed whether *TRANK1-*rs9867455 and *VWA5B2*-rs9837978 are associated with the expression of nearby genes. Open Targets reported rs9867455 as a significant eQTL (*P* < 1 × 10^−6^) for *LRRFIP2*, *ITGA9*, *GOLGA4*, *MLH1*, and *TRANK1* across blood or other tissues. *LRRFIP2, GOLGA4* and *TRANK1* were also nominated in the multi-ancestry brain eQTL data. Open Targets reported rs9837978 as a significant eQTL for *AP2M1*, *ABCF3*, *VWA5B2*, *ALG3*, *ABCC5*, *DVL3*, and *CLCN2*. *AP2M1*, as well as two additional genes (*EIF2B5* and *ECE2*) were nominated in the multi-ancestry brain eQTL data.

To prioritize susceptibility genes with expression effects on ADD risk, we performed summary-based Mendelian Randomization (SMR) to infer whether expression of the eQTL-nominated genes is causal for AD. More details regarding the purpose and methods used to perform SMR can be found in the “Functional inferences” section of the “Online Methods”. At the *TRANK1-*rs9867455 locus, *TRANK1*, *LRRFIP2*, *GOLGA4*, and *ITGA9* were significant in our SMR results for affecting AD risk via expression across multiple tissue types. The strongest associations in cortex tissue were seen with *TRANK1* and *LRRFIP2*. The GWAS signal at the *TRANK1-*rs9867455 locus colocalized most strongly with *TRANK1* expression in cortex tissue (*R*^2^ = 0.52; Fig. S3). At the *VWA5B2-*rs9837978 locus, *VWA5B2*, *AP2M1*, *ABCF3*, *ALG3*, *EIF2B5*, *DVL3*, *CLCN2*, *ABCC5* were significant in our SMR results. The strongest associations in brain tissues were seen in *ABCF3*, *ALG3* and *EIF2B5*, although colocalization between the GWAS signal and these eQTLS were not very strong (*R*^2^ < 0.5; Fig. S4). For more details on directionality of these associations, see Table S3.

### Fine-mapping

A total of nine loci outside of the *APOE, MAPT*, and major histocompatibility complex (MHC) regions were fine-mapped to a credible set of ≤ 2 SNPs with a combined posterior probability (PP) of 99% (Table 2 and Figs. S5, 6). The *MHC* and *MAPT* regions were excluded from fine-mapping due to a complex haplotype structure across populations [28, 29] and known haplotype inversions [30], respectively. Five of these loci were previously fine-mapped with PP > 0.8 in large GWAS of European populations (Fig. S5; *BIN1*-rs6733839; *INPP5D*-rs10933431; *ECHDC3*-rs7912495; *APH1B*-rs117618017; *ABCA7*-rs12151021) [31, 32]. Four additional ADD loci with 1–2 variants in their 99% credible sets have not been previously fine-mapped (Fig. S6a-b, d-e; *RHOH*-rs2245466; *CTSB*-rs1065712; *FAM157C/PRDM7*-rs56407236; *GRN*-rs5848). Interestingly, *GRN* is also a candidate risk locus for frontotemporal dementia (FTD) [33] and Parkinson’s disease (PD) [34, 35]. More than 70 pathogenic variants in *GRN* have been linked to familial FTD [33]. While *GRN* mutations do exist in sporadic forms of FTD, we were unable to find publicly available FTD GWAS summary statistics with a significant association at this locus. Using publicly available PD GWAS summary statistics [34], we compared the regional genetic correlations between PD and ADD at both *GRN* and *CTSB*, another candidate risk locus for PD. LocusCompare plots show low genetic correlation between ADD and PD at both loci (*GRN*, *R*^2^ = 0.36; *CTSB*, *R*^2^ = 0.0048), which may indicate that distinct causal variants drive the associations (Fig. S7). One additional locus with a credible set size >2 was fine-mapped to a single SNP with PP > 0.8 (Fig. S6c; *SLC24A4*-rs12590654, PP = 0.91, *n* = 32 in 99% credible set). In addition, two SNPs with a PP ≥ 0.3 were annotated as missense variants (Fig. S8; *MS4A6A*-rs7232, PP = 0.54, *n* = 4 in 99% credible set; *SHARPIN*-rs34674752, PP = 0.30, *n* = 5 in 99% credible set). Notably, our fine-mapping analysis did not replicate *SORL1*-rs11218343, which has been previously fine-mapped with a PP > 0.999 in two large European studies [31, 32], likely due to a different regional architecture in the East Asian population as has been previously reported (Fig. S9) [36]. All 99% credible sets are provided in Table S4.

**Table 2:** Fine-mapping results for all SNPs with a posterior probability (PP) > 0.8.

Locus	SNP	Chromosome	Position	Effect allele	Reference allele	Novel fine-mapping	Nsnps_in_credible_set	Posterior probability	P, MR-MEGA	P, ancestry heterogeneity	chisq, ancestry (% total)	I2
*BIN1*	rs6733839	2	127892810	T	C	No	1	1	5.74E−92	3.13E−01	14.270	58.010
*INPP5D*	rs10933431	2	233981912	C	G	No	1	1	1.63E−16	7.90E−02	41.675	59.470
*RHOH*	rs2245466	4	40198846	C	G	Yes	1	1	3.82E−09	9.31E−01	0.128	49.160
*CTSB*	rs1065712	8	11702122	C	G	Yes	1	1	6.42E−09	7.60E−01	4.551	0.000
*ECHDC3*	rs7912495	10	11718713	C	G	No	1	1	1.35E−13	6.02E−01	48.894	0.000
*SLC24A4*	rs12590654	14	92938855	A	G	Yes	32	0.910	6.56E−16	3.37E−02	63.950	57.450
*APH1B*	rs117618017	15	63569902	T	C	No	2	0.965	3.63E−20	4.98E−01	13.608	10.950
*FAM157C/PRDM7*	rs56407236	16	90170095	A	G	Yes	1	1	7.55E−13	1.33E−01	63.528	15.600
*GRN*	rs5848	17	42430244	T	C	Yes	1	1	1.87E−15	3.22E−01	20.238	38.050
*ABCA7*	rs12151021	19	1050874	A	G	No	1	1	3.62E−32	3.23E−01	34.721	0.000

Fine-mapped SNPs were considered novel if they were not previously fine-mapped with PP > 0.8 in two recent European-focused studies by Wightman et al. and Schwartzentruber et al. [31, 32].

### Heterogeneity analysis

**Fig. 3:**
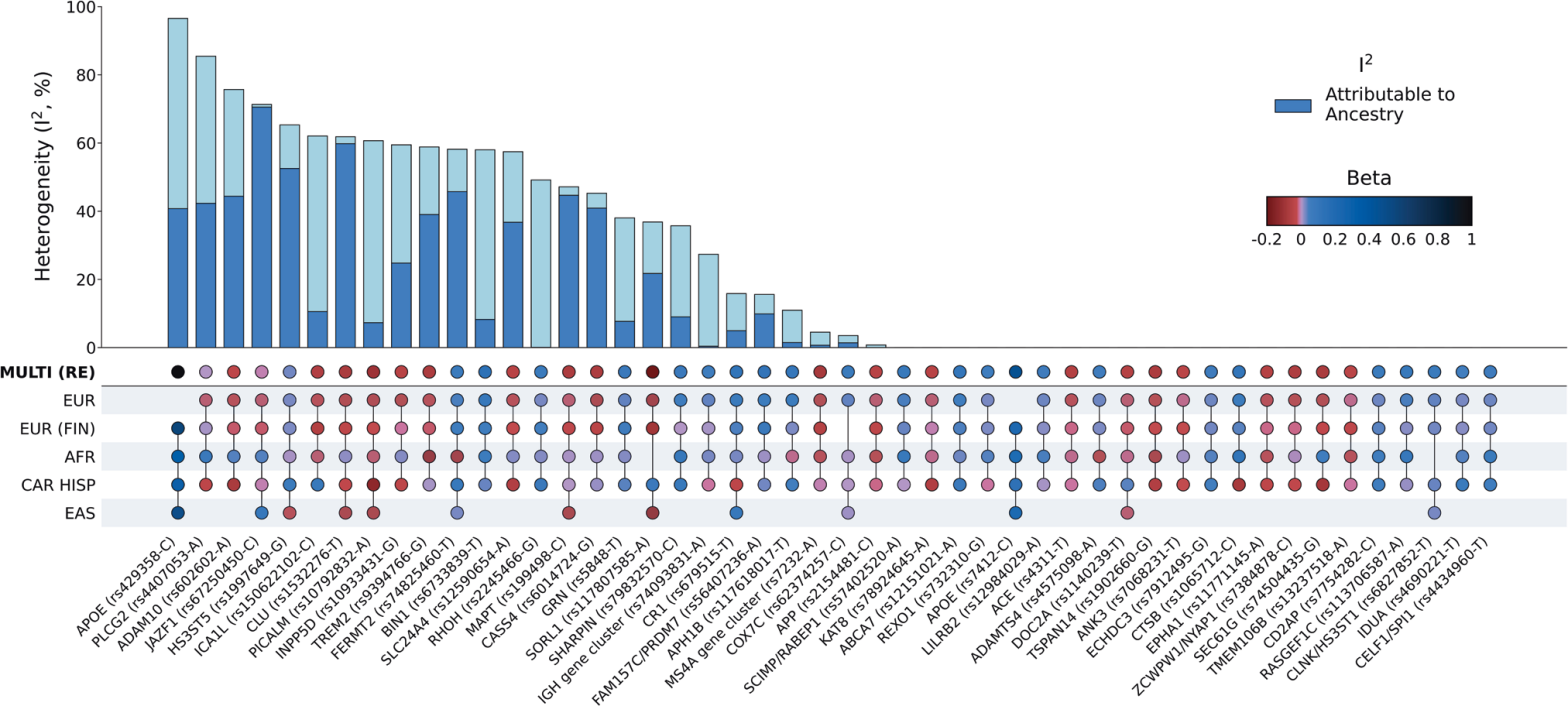
Graphical summary of heterogeneity at ADD genetic risk loci. Lead SNPs were derived from MR-MEGA using maximal LD blocks, apart from *APOE* rs429358 and rs7412. Both *APOE* SNPs were absent in summary statistics from the most recent European-ancestry ADD GWAS. Aggregate effects were estimated using a random effects model since MR-MEGA assumes that effects differ across populations. Allelic effect heterogeneity that is attributable to genetic ancestry was estimated using Cochran’s Q statistics for ancestral and residual heterogeneity from the meta-regression (“Online Methods”).

We observed significant heterogeneity (*I*^2^ > 30%) at 19 of the 48 loci that reached genome-wide significance (*P* < 5 × 10^−8^) in MR-MEGA (Table S2a), which does not include the additional loci (including 2 novel) identified by the fixed and random effects analyses (Table S2b, c). Several factors can account for the observed heterogeneity, such as differences in study design, geographical region, and diagnostic accuracy. We estimated the proportion of heterogeneity that is attributable to genetic ancestry using MR-MEGA (see “Assessment of allelic effect heterogeneity” in the “Online Methods”) and observed that at least 50% of the heterogeneity was attributable to genetic ancestry at 10 of these loci (Figs. 3 and S10). We also assessed heterogeneity at lead SNPs from the most recent European GWAS [4] and found that 37% of the lead SNPs tested presented significant heterogeneity (*I*^2^ > 30%), of which 48% were primarily attributable to ancestry (Table S5). Five of the fine-mapped SNPs also showed significant heterogeneity (*I*^2^ > 30%), of which only *SLC24A4* showed heterogeneity that was primarily attributable to genetic ancestry (Table 2).

The genetic polymorphisms rs7412 and rs429358 that form the *APOE* e2/e3/e4 alleles presented very different allelic heterogeneity. Consistent with previous studies [7, 37], we observed an attenuated signal at *APOE*-rs429358, which determines the *APOE*-e4 allele, among the cohorts of African Americans and Caribbean Hispanics (Fig. S11a). *APOE*-rs429358 had the highest heterogeneity (*I*^2^ = 96.54) of the SNPs tested with ~42% attributable to genetic ancestry, although these polymorphisms were not available to test in the most recent European-ancestry AD GWAS [4]. In contrast, *APOE*-rs7412, which determines the *APOE*-e2 allele, did not present any heterogeneity (Fig. S11b; *I*^2^ = 0). Complementary to standard GWAS association tests, we also generated *P* values representing heterogeneity of effect estimates attributable to genetic ancestry in the multi-ancestry meta-regression and observed the strongest signal near *APOE* (Fig. S12). Additionally, we observed strong evidence of ancestry-related heterogeneity (*P*_HET_ < 1e−6) near *SORL1*, as well as *PAPOLG*, *AC026202.5*, and *snoU13* which did not meet genome-wide significance in the association results. LocusZoom and beta-beta plots of these loci suggest that non-European populations primarily drive these association signals, and there are likely discordant effects across populations (Fig. S13).

### Polygenic risk scoring

**Fig. 4:**
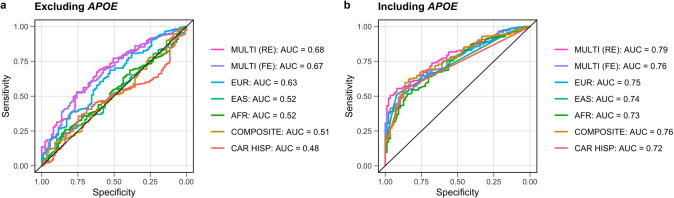
Graphical summary of genetic risk scores. These genetic risk scores were derived from multi-ancestry and ancestry-specific risk estimates, then applied to an admixed Colombian cohort to evaluate significance and predictive power. The European-based (EUR) PRS was derived from a fixed effect meta-analysis of the summary statistics from Bellenguez et al. and FinnGen used in the meta-GWAS. **a**, **b** The maximal AUCs for each genetic risk score with color coding to delineate the source of the risk estimates for scores excluding and then including *APOE-e4* variants. *P* value thresholds that correspond to the maximal AUCs are shown in Table S6.

We tested the performance of the multi-ancestry fixed and random effects models and each of the GWAS from single ancestral populations in a Colombian cohort of AD cases (*n* = 281) and neurologically normal controls (*n* = 87). This cohort is an admixture of three ancestral populations, with European substructure making up the highest proportion of global ancestry (mean of 64%, SD = 15%), followed by Indigenous American (mean of 27%, SD = 11%), and African (mean of 9%, SD = 11%). Colombian samples were used to test PRS applicability as they were a population not represented in the meta-analyses. While the Caribbean Hispanic cohort included in the meta-analyses is also three-way admixed, this cohort likely has a lower Indigenous American proportion and higher African ancestry proportion than the Colombian cohort [38]. Single-ancestry PRS performed worse than multi-ancestry random-effects-derived PRS in terms of area under a receiver operating characteristic curve (AUC). We observed maximal AUCs of 79% and 68% including and then excluding *APOE* variants in this population, and 75% and 63% for the European-derived PRS (Fig. 4; Table S6). Non-European AUCs tended to improve with increasing sample size (Fig. 4), suggesting that the composite score, combining ancestry-specific PRS by population weights, may have performed as well or better than the random-effects-derived PRS if the component GWASs from underrepresented populations were better powered.

## Discussion

We performed a large, genome-wide meta-analysis of ADD across five datasets, representing four super-populations. By leveraging data from multiple ancestry groups, we replicated 66 known ADD loci and identified two novel risk loci on chromosome 3 (Tables S2a–c). These novel loci reached genome-wide significance using the fixed and random effects models, but were sub-significant using MR-MEGA as this model is not as well-powered to detect loci with homogenous allelic effects [25].

The first novel locus identified in this study is near *TRANK1*, which encodes tetratricopeptide repeat and ankyrin repeat containing 1. *TRANK1* is associated with DNA- and ATP-binding and DNA repair and is highly expressed in brain tissue [39, 40]. Previously, *TRANK1* has been cited as a robust risk locus for both schizophrenia (SZ) [41–43]. and bipolar disorder (BD) [39, 44, 45], although subtype analyses suggest that this signal is primarily driven by the most heritable subtype, BD I, which is genetically correlated with SZ [46]. Notably, BD has also been shown to increase risk for AD, with the two sharing significant genetic overlap [47]. LocusCompare plots show a modest correlation between our random effects meta-analysis of ADD with BD I (*R*^2^ = 0.46) and SZ (*R*^2^ = 0.23) GWAS at this locus (Figs. S14 and 15). We also observed moderate LD (*R*^2^ = 0.44, 1000 Genomes EUR) between the lead SNP identified in our study (rs9867455) and the lead SNP identified in the BD I GWAS (rs9834970), indicating potential cross-disease overlap for this locus.

Previous SMR analysis of the *TRANK1* region in BD suggested that both *TRANK1* and *GOLGA4* may be susceptibility genes [48]. *TRANK1* expression is decreased in both BD and AD, and decreased expression of *TRANK1* was found to alter the expression of genes related to neuronal development and differentiation [47]. Altered neurogenesis has been implicated in human AD brains and AD rodent models [48]. Previous studies have suggested that *TRANK1* may be involved in blood brain barrier permeability changes and neuroinflammation, both of which may be relevant to neurodegeneration [48, 49]. Interestingly, Kunkle et al. nominated *TRANK1* through a gene-based analysis conducted in an African American population, but not through the single SNP association testing included in this study [6]. A combination of eQTL and SMR nominated *TRANK1*, *LRRFIP2*, *GOLGA4*, and *ITGA9* as potential genes underlying this SNP association, with the strongest associations in cortex tissue seen with *TRANK1* and *LRRFIP2* (Table S3). *LRRFIP2* encodes LRR binding FLII interacting protein 2, which regulates Toll-like receptor 4 (TLR4) and can downregulate the *NLRP3* inflammasome. TLR4 can induce microglial amyloid-β clearance in the brain in early stages of AD but can later induce an inflammatory response, suggesting that disruptions to *LRRFIP2* may affect AD pathology in patients [50].

The second locus is nearest to *VWA5B2*, which encodes von Willebrand factor A domain-containing protein 5B1. Von Willebrand factor (VWF) is a glycoprotein that facilitates blood clotting at areas of injury. High VWF is associated with short-term risk of dementia, possibly due to the increased risk of blood clots restricting blood flow in the brain [51]. Interestingly, *VWA5B2* was found to be downregulated in AD patients, and other variants in *VWA5B2* have been linked to decreased mean hemoglobin concentration [12, 52]. Low hemoglobin, or anemia, has been linked to an increase in risk for AD [53]. This information seems to further implicate the involvement of the vasculature system with AD, complementing previous studies such as those investigating traumatic brain injury [54]. Whether *VWA5B2* has biological implications on risk for AD needs to be further investigated. The lead variant rs9837978 does not lie within any of the nearby genes at this locus, but eQTL and SMR evidence for this variant nominated eight nearby genes including *VWA5B2* (Table S3). The strongest SMR association in brain tissue is seen with *ABCF3*. The ABCF3 protein is a member of the ATP-binding cassette (ABC) superfamily, all of which transport a variety of substrates across intra- and extracellular barriers [55]. Members of the ABC A subfamily, such as *ABCA7* and *ABCA1*, have previously been nominated as AD risk genes [4]. *ABCF3* is a unique family member in that it lacks a transmembrane domain but has been nominated as a candidate of TLR signaling, similar to *LRRFIP2* [56]. In addition to inducing inflammatory responses, TLRs can affect microglial activity, synaptic plasticity, and tau phosphorylation, providing additional evidence to their potential importance in AD pathology [57]. Additionally, downregulation of *ABCF3* has been associated with an increase in viral load after infection by a flavivirus, specifically the West Nile virus which has been linked to long-term neurological problems and dementia [58, 59]. It’s plausible that in the presence of viral infection, changes in *ABCF3* expression may affect immune response and inflammation, two processes that play a role in the amyloid cascade hypothesis [60].

Future studies will be required to further disentangle the potential roles of the nominated genes in the context of ADD risk. Although genome-wide summary statistics were unavailable for replication, we attempted to replicate the lead SNP *VWA5B2*-rs9837978 in a small East Asian cohort [20]. However, this SNP demonstrated an opposite direction of effect and the replication dataset was underpowered to detect an association (Fig. S16). Assuming a disease prevalence of 2%, MAF of 0.1642 (gnomAD v3.2.1 East Asian), and a nominal significance threshold of 0.05, we had ~80% power to detect genotype relative risks ≥1.245 but our odds ratio for this allele, which is generally an overestimate of risk [61], was 1.05. Replication in larger and more diverse cohorts is warranted in future studies. Further, the disparity seen at points between the results on Open Targets, which consists of largely European data, and the multi-ancestry eQTL results for nominated genes also highlights the need for more multi-modal reference data including diverse ancestries. However, it is also possible that there could be different mechanisms underlying disease risk conferred by the implicated loci across different populations.

Our study highlights the utility of multi-ancestry datasets at uncovering putative mechanisms that contribute to ADD. Fine-mapping at several known ADD loci was better resolved using the multi-ancestry meta-regression compared to previous efforts in European populations. For example, fine-mapping near *RHOH*, *CTSB* and *FAM157C/PRDM7* nominated variants that are located in untranslated regions that were not well-resolved in European-focused studies. Variants in the 3′UTR region can impact translation or protein stability, and transcription binding can be impacted by variants in the 5′UTR region. Additionally, *GRN*-rs5848 is associated with circulating progranulin levels and decreased *GRN* expression has been implicated in several neurodegenerative diseases, including AD and FTD [35, 62, 63]. In contrast to previous studies in European populations, the *SORL1* locus was not resolved to a single putative causal SNP. Lead SNPs in both the European (*SORL1*-rs11218343) and East Asian (*SORL1*-rs117807585) GWAS are more common among East Asians compared to all other populations in the Genome Aggregation Database v2.1.1 (rs11218343: AF_EAS_ = 0.30, AF_EUR_ = 0.039; rs117807585: AF_EAS_ = 0.22, AF_EUR_ = 0.020). It is possible that alternative fine-mapping approaches that allow for multiple causal variants per locus will provide greater insight into the *SORL1* locus.

At the *MS4A* gene cluster, multi-ancestry fine-mapping resolved the signal to a credible set of five variants, with a common missense variant (rs7232, PP = 0.54) and an intergenic variant nearest *MS4A4A* (rs1582763, PP = 0.45) that are in moderate LD (*R*^2^ = 0.55, 1000 Genomes all populations) having the highest probability of causality (Fig. S8). *MS4A4A* and/or *MS4A6A* modulate soluble TREM2 (sTREM2) in cerebrospinal fluid (CSF), which is correlated with AD progression. Previous studies have shown that rs7232 is associated with *MS4A6A* gene expression and CSF sTREM2 [64, 65], while rs1582763 is a cis-eQTL for *MS4A4A* and *MS4A6A* [66]. Conditional analysis of CSF sTREM2 levels in this region have pointed to two independent signals represented by rs1582763 and rs6591561 (MS4A4A p.M159V) [66]. Therefore, a fine-mapping approach that allows for multiple causal variants may be more appropriate for this region.

In addition to highlighting genetic risk factors that are shared across populations, our results also highlight ADD loci with significant heterogeneity that may reflect variation in effect sizes, allele frequencies or interaction(s) with environmental risk factors that vary by ancestral group. For example, we observed the strongest evidence of heterogeneity at *APOE*-rs429358. Around 42% of the heterogeneity at this allele was attributable to genetic ancestry, while the remaining heterogeneity may reflect other sources of variation such as imputation accuracy since this allele is rarely assayed successfully on genotyping arrays. At *JAZF1*-rs67250450 and *CLU*-rs1532276, we observed the strongest evidence of ancestry-related heterogeneity, both of which are most common among individuals of East Asian ancestry and showed the strongest effects in this population (Fig. 3). We also observed significant ancestry-related heterogeneity at *SORL1* and *TREM2*, which have been previously shown to harbor population-specific risk variants [67, 68]. Given that our analyses focused on common variation, the effects of rare heterogeneous variants (e.g. *ABCA7*-rs115550680, which has comparable effects to *APOE*-rs429358 among African Americans [69]) may not have been fully captured.

While this study marks progress towards assessing genetic risk of ADD across multiple populations, we acknowledge several limitations. First, we recognize that the magnitude of clinical and pathological diversity among ADD cases is extensive. The diagnostic inaccuracy rate of AD likely differs across studies and populations [70, 71] both early-onset forms of AD and other pathologies such as frontotemporal and lewy body dementias. This clinicopathological heterogeneity is further exacerbated by the inclusion of proxy-ADD cases in the Bellenguez et al. study, a study which comprised the majority of European samples included in our analysis. While proxy-ADD cases may introduce more variability than a clinical or pathological diagnosis of AD, prior studies have demonstrated strong genetic correlation between proxy-ADD and AD (rg = 0.81), further supporting the use of this data [72]. Additionally, despite the phenotypic heterogeneity in the Bellenguez et al. study, the utility of ADD GWAS are supported by genes with well-defined involvement in relevant molecular pathways. For example, the *APP* locus was first identified at genome-wide significance in Bellenguez et al. (*P* = 1.02 × 10^−9^) and we found a stronger level of significance in our random effects meta-analysis (*P* = 8.1 × 10^−12^, *I*^2^ = 0.79). This locus is likely driven by underlying AD pathology due to its role in the formation of amyloid-β and its previous implications in both LOAD and EOAD [73, 74]. Although disease subtype-specific conclusions that can be made from our analysis are limited by the diagnostic criteria of the included GWAS, similar analyses can be applied as larger datasets with high phenotypic specificity become available.

Additionally, although MR-MEGA is a useful tool for fine-mapping and ancestral heterogeneity estimation, the software requirements of population overlap (*K* > 3) often result in reduced variant sets after study level quality control. This can bias fine-mapping results as we reduce the potential resolution on local haplotypes, and usually necessitates the inclusion of at least one of the larger European-focused studies. In our case, previous European and Finnish studies served as the backbone of our meta-GWAS. We did not replicate previous fine-mapping at *NCK2*, *TREM2* and *RNF223* from European-focused studies since study level quality control included filtering for common (MAF > 1%), biallelic SNVs due to potentially poor imputation and general low power for rare variants across ancestral groups. We acknowledge that variants with a minor allele frequency <1% in one or more populations, as well as indels and structural variants, may contribute to the observed associations. While less stringent QC may have allowed us to detect more variants, we used a more conservative approach to accommodate the smaller sample size of non-European GWAS and the need for MR-MEGA SNPs to be present in at least four studies. Future work should include imputation using diverse reference panels from long read sequence data specific to ADD to improve genomic coverage and provide insights into structural variation that may be population specific.

In addition, the number of axes of genetic variation (T) in MR-MEGA is restricted to *T* ≤ K-2, where *K* is the number of input GWAS. The East Asian GWAS from Shigemizu et al. used in our meta-GWAS tested less than half as many SNPs as the others (Table S1), limiting the meta-regression to a single axis of genetic variation (PC0) at SNPs that overlap the remaining GWAS (*K* = 4). Including a larger number of input GWAS from underrepresented populations will likely improve the heterogeneity estimates outlined in this study and are worth pursuing when such data become available.

While our study is inclusive, due to data availability and the European-dominated nature of genetic research, European-ancestry individuals make up approximately 85% of cases and the discovery efforts here maintain a baseline level of Eurocentric bias. Despite this bias, our random-effects-derived PRS including *APOE* variation achieved a higher maximal AUC of 79% in an independent admixed Colombian cohort compared to 75% achieved by the European-based PRS. Additionally, while our novel method of creating a composite PRS model that leverages admixture percentages is a potentially promising approach for assessing ADD risk across ancestrally heterogeneous and/or admixed cohorts, its performance relies on sufficient sample sizes and global genetic representation. As larger scale GWAS for multiple continental “super populations” continue to become available, we believe this method of tuning PRS to an individual’s genetic admixture could have utility in a precision medicine context. Reducing the Eurocentric bias in AD genetics research will require the harmonization and refining of diagnosis in non-European research sites that serve communities with unique cultural and logistic concerns for participation in research. Overall, our study provides a critical framework for future ADD meta-analyses. It is our hope to improve representation in ADD genetic studies in the future, increasing the balance between European and well-powered non-European cohorts.

## Online methods

### Existing GWAS studies

Summary statistics from Bellenguez et al. 2022 were accessed through the National Human Genome Research Institute-European Bioinformatics Institute GWAS catalog under accession number GCST90027158 in May 2022. Summary statistics from FinnGen Release 6 were accessed at https://www.finngen.fi/en/access_results in April of 2022. Summary statistics from Kunkle et al. [6] were accessed through NIAGADS (https://www.niagads.org/) under accession number NG00100 in April of 2022. Summary statistics from Shigemizu et al. [19] were accessed through the National Bioscience Database Center (NBDC) at the Japan Science and Technology Agency (JST) at https://humandbs.biosciencedbc.jp/en/ through accession number hum0237.v1.gwas.v1 in April of 2022. All summary statistics were aligned to GRCh37 and cleaned to remove indels, multi-allelics and rare variants (MAF < 1%) prior to multi-ancestry analysis.

### Caribbean Hispanic GWAS

Data from the Columbia University Study of Caribbean Hispanics and Late Onset Alzheimer’s disease were accessed via application to dbGaP accession number phs000496.v1.p1 in April of 2022. Samples were filtered to keep unrelated individuals without missing values for AD affection status, age, study category, education, and a missing call rate <0.02. Principal component analysis was performed on a combined dataset of study subjects and HapMap was used as a reference to identify potential outliers. Controls with a family history of dementia were removed to ensure that potential proxy-ADD cases were not present in the control group. Variant QC included exclusion filters for monomorphic SNPs, variants with MAF <1%, missingness rates >2%, sex differences in allelic frequency ≥0.2 and heterozygosity >0.3, duplicate SNPs, Hardy–Weinberg Equilibrium (HWE) *P* value <1 × 10^−4^, and >1 discordant calls or Mendelian errors. All variants with a significant frequency mismatch (*χ*^2^ > 300) with the TOPMed reference panel were removed prior to imputation.

As increasing age is the most significant risk factor for ADD, age matching is commonly used to control for differences in age distributions between cases and controls. While we did not perform case-control age matching to maximize sample size, the distribution of ages between cases and controls largely overlaps and we include age as a covariate in our GWAS analysis. A demographic summary table detailing age, *APOE*-e4 status, sex for the Caribbean Hispanic cohort is provided in Table S7.

Using PLINK v1.9 [24], we evaluated the association between AD and imputed genotypes via logistic regression on allele dosages with imputation quality >0.3, adjusting for sex, age (age at disease onset for cases, age at last evaluation for controls), education, study category, and the first 10 principal components (PCs). Study category denotes subcategories within the Caribbean Hispanic dataset (individuals are from the United States, Puerto Rico and the Dominican Republic) and is included to account for potential batch effects.

### Meta-analysis and fine-mapping

#### Meta-analysis

Three models were used to conduct multi-ancestry meta-analyses. Fixed effect and random effects models were performed using PLINK v1.9, while a separate analysis was performed using MR-MEGA v0.2 [25]. PLINK v1.9 was preferred over METAL due to its capacity to perform fixed and random effects analyses in parallel. A random effects model provides a more conservative framework which allows each study to have unique effects, as can be expected in different populations. MR-MEGA was also employed since it is well-powered to detect associations at loci with allelic heterogeneity. MR-MEGA models allelic effects as a function of axes of genetic variation that are derived from the input GWAS summary statistics. This method can result in reduced variant sets since it requires that variants have sufficient overlap between the input datasets (*K* > 3), where K is the number of input GWAS, in contrast to both the fixed and random effects models implemented in PLINK v1.9 which were limited to *K* > 2 to accurately quantify heterogeneity.

The European and Finnish European GWAS were included separately in all multi-ancestry meta-analyses to account for finer-scale differences in allele frequencies. To determine the optimal number of PCs needed to distinguish cis- and multi-ancestry ADD summary statistics using MR-MEGA, we visually inspected pairwise PC plots generated using all five GWAS referenced in Table S1. We observed adequate separation between the Caribbean Hispanic, European, African American, and East Asian GWAS using the first two meta-regression PCs (Fig. S17). To increase the variant set, we also ran MR-MEGA separately for each combination of four input GWAS. A single axis of genetic variation (*T* = 1) was included in this analysis since this is the maximum allowable given the constraints of the model (*T* ≤ *K*−2). Summary statistics were aggregated to maximize the effective sample size for each variant.

#### Defining associated loci

FUMA was used to find maximal LD blocks around loci that reached *P* < 5 × 10^−8^ in the specified multi-ancestry meta-analysis. LD blocks of independent significant SNPs (*R*^2^ > 0.3, 1000 Genomes all populations) were merged into a single genomic locus if the distance between LD blocks was less than 250 kb. These loci were compared to the previous GWAS by Bellenguez et al. [4] and Open Targets to assess whether these regions were known to be associated with ADD. These genomic intervals were also used as inputs for fine-mapping as described below.

#### Fine-mapping

Fine-mapping was performed using approximate Bayes’ factors in favor of association from the meta-regression model implemented in MR-MEGA. Posterior probabilities (PP) were calculated using single-SNP Bayes factors and credible sets were generated for each locus (with genomic intervals defined as described above) until the cumulative PP exceeded 99%. All SNPs in the 99% credible sets were annotated with VEP (http://grch37.ensembl.org/Homo_sapiens/Tools/VEP) using default criteria to select one block of annotation per variant (Table S4).

### Assessment of allelic effect heterogeneity

Allelic effect heterogeneity between studies was assessed for all lead SNPs reaching genome-wide significance (*P* < 5 × 10^−8^) in the meta-regression, implemented in MR-MEGA. The meta-regression model derives axes of genetic variation from pairwise allele frequency differences between the input GWAS. Heterogeneity is then partitioned into (1) ancestry-related heterogeneity that is correlated with the axes of genetic variation and (2) residual heterogeneity that is likely due to other factors such as diagnostic accuracy, study design (e.g. covariate adjustments, phenotype definition, imputation quality, inclusion of proxy-ADD cases) and/or geographical region. Total heterogeneity at each index SNP was quantified using the *I*^2^ statistic in PLINK v1.9 to avoid bias due to sample size for SNPs not tested in the large European studies. The *I*^2^ statistic describes the proportion of variation in effect estimates that is due to heterogeneity. We considered SNPs with an *I*^2^ > 30% as having significant heterogeneity since this suggests at least moderate variation in allelic effects [75]. The percentage of this heterogeneity that is attributable to genetic ancestry was then calculated using Cochran’s Q statistics for ancestral and residual heterogeneity from the meta-regression (Eq. 1; ANC: ancestry, RESID: residual).

1 $$\%\,\,{{{{{\rm{Heterogeneity}}}}}}_{{{{{\rm{ANC}}}}}}=Q_{{{{{\rm{ANC}}}}}}/(Q_{{{{{\rm{RESID}}}}}}+Q_{{{{{\rm{ANC}}}}}})\times 100\%$$

### Functional inferences

To prioritize genes underlying the two novel loci, we first looked at public eQTL data to determine whether the GWAS-identified lead variants are eQTLs for nearby genes. This allowed us to cast a wide net of potential regional genes of interest. We employed Open Targets for this effort, which shares eQTL results for variants from blood, brain, and a wide array of tissues from multiple public eQTL datasets [26]. We additionally investigated a multi-ancestry brain eQTL dataset [27] which was not available on Open Targets at the time of publication. We considered the lead variants as significant eQTLs for a gene if they passed the significance threshold of *P* < 1 × 10^6^, which has been shown to correspond to a genome-wide false discovery rate (FDR) of 5%, although we do acknowledge this may be overly conservative in our regional analyses [76].

Once we had nominated potential genes for which our lead variants were significant eQTLs, we used summary-based Mendelian Randomization (SMR) to make functional inferences as to whether the disease risk SNPs in these regions mediate gene expression. We integrated summary-level data from the most recent ADD GWAS [4] with data from multiple eQTL studies in different tissues using the SMR method [77]. SMR uses summary statistics to determine if an exposure is associated with a trait through a shared casual variant. MR can be used to mimic a randomized controlled trial, as having a variant that increases or decreases expression of a gene may be comparable to life-long treatment with a drug targeting the encoded protein of that gene. [78] For example, if SNP A affects gene B expression (the exposure), and SNP A is also associated with ADD risk (the outcome), you can infer the causal effect of the expression of gene B on ADD risk.

We limited our results to the genes that were prioritized by our eQTL search and considered a gene significant for expression effect on a disease if it passed an FDR-adjusted SMR significance threshold of *P* < 0.05 and a HEIDI threshold of *P* > 0.01. Filtering for a HEIDI P-value of this magnitude helps to remove associations that are likely due to polygenicity and have violated the central assumptions of SMR. Finally, we assessed the colocalization between the SMR-nominated genes in brain tissues and the multi-ancestry random effects GWAS using LocusCompare [79].

### Polygenic risk scoring

#### PRS application cohort

Whole genomes from the Colombian population were accessed from “The Admixture and Neurodegeneration Genomic Landscape” (TANGL) study and quality controlled as previously described [67]. The TANGL cohort was further quality controlled in PLINK v1.9 to remove carriers of pathogenic variants for mendelian forms of dementia, as well as related individuals for a final cohort of 281 cases and 87 controls.

#### Pre-PRS variant alignment

Base summary statistics were pruned with the MungeSumStats R package [80] to remove multiallelic variants, align reference alleles to build GRCh37, and adjust weights for the appropriate reference alleles. The target TANGL cohort was also filtered to keep only biallelic variants and aligned to the same reference using PLINK v2.0.

#### PRS method

Polygenic risk score (PRS) analyses can be used to estimate an individual’s genetic liability to a phenotype by calculating the sum of risk allele effect size weights for an individual. Weights for the PRS were obtained from β estimates generated from multi-ancestry fixed and random effects meta-analyses as well as from individual ancestry summary statistics. PRS analyses were conducted using PRSice v2.3.5 including variants with minor allele frequency >5%, genotype missingness <10%, sample missingness <10%, and HWE *P* value <1 × 10^−6^. The *APOE* region (with ranges defined by FUMA as described previously) was excluded prior to variant clumping. For PRS analyses including *APOE*, the genetic polymorphisms rs7412 and rs429358 were added to the QC’d summary statistics prior to variant clumping. β estimates for the *APOE* polymorphisms were not available in the Bellenguez et al. summary statistics [4] and therefore European-ancestry estimates were taken from another recent ADD GWAS by Schwartzentruber et al. [32].

Variants were clumped in each 500 kb window with the index SNP at the center, an *r*^2^ threshold of 0.3, and a clump *P* value threshold of 1. Sex, age, and the first 5 PCs were used as covariates in the PRS analysis. PCs were generated from non-imputed genotype data using FlashPCA [81]. Variants with a MAF <1%, genotype missingness <10%, sample missingness <10%, and HWE *P* values <5 × 10^6^ were excluded using PLINK v1.9. The remaining variants were pruned with a 1000-kb window, a 10-SNP shift per window and an *r*^2^ threshold of 0.02 prior to PC calculation. PRS analysis was performed at select P-value thresholds to determine the best fit model (*P* = 5 × 10^10^, 5 × 10^−9^, 5 × 10^−8^, 5 × 10^−7^, 5 × 10^−^^6^, 5 × 10^−5^, 5 × 10^−4^, 5 × 10^−3^, 5 × 10^−2^). To assess the performance of each model, receiver operator characteristic curves were created using the pROC library in R for the best fit model from each analysis as shown in Table S6. Since MR-MEGA does not provide standard effect estimates per SNP, an additional “composite” ROC curve was generated through a linear combination of each super population to provide a comparison to the conservative random-effects-based PRS model. Each PRS was weighted by its associated admixture population percentage, previously determined in the TANGL cohort for each individual (Eq. 2; AFR: African American, EUR: European (including Finnish), EAS: East Asian, NAT: Native American) [67]. Given the population history and similarities in haplotype structure between the East Asian and Native American populations, Native American admixture proportions were used to weight the East Asian PRS [82, 83].

2 $${{{{{\rm{PRS}}}}}}_{{{{{\rm{composite,i}}}}}}= \;{{{{{\rm{PRS}}}}}}_{{{{{\rm{AFR,i}}}}}}{\times}\%_{{{{{\rm{AFR,i}}}}}}+{{{{{\rm{PRS}}}}}}_{{{{{\rm{EUR,i}}}}}}\\ \times\%_{{{{{\rm{EUR,i}}}}}}+{{{{{\rm{PRS}}}}}}_{{{{{\rm{EAS,i}}}}}}{\times}\%_{{{{{\rm{NAT,i}}}}}}$$

### Supplementary information

Supplemental Material

Supplemental Tables

## Data Availability

Summary statistics from this study will be available to browse and download via our collaboration with the Broad’s Neurodegenerative Disease Knowledge Portal (https://ndkp.hugeamp.org/dinspector.html?dataset=Lake2023_AD_Mixed).

## Code availability

Code is available on the CARD GitHub (https://github.com/NIH-CARD/MA_MA_meta).

## References

[1] Tanzi RE, Bertram L (2001). New frontiers in Alzheimer’s disease genetics. Neuron..

[2] Holland D, Frei O, Desikan R, Fan C-C, Shadrin AA, Smeland OB (2021). The genetic architecture of human complex phenotypes is modulated by linkage disequilibrium and heterozygosity. Genetics..

[3] Zhang Q, Sidorenko J, Couvy-Duchesne B, Marioni RE, Wright MJ, Goate AM (2020). Risk prediction of late-onset Alzheimer’s disease implies an oligogenic architecture. Nat Commun.

[4] Bellenguez C, Küçükali F, Jansen IE, Kleineidam L, Moreno-Grau S, Amin N (2022). New insights into the genetic etiology of Alzheimer’s disease and related dementias. Nat Genet.

[5] Hou K, Bhattacharya A, Mester R, Burch KS, Pasaniuc B (2021). On powerful GWAS in admixed populations. Nat Genet.

[6] Kunkle BW, Schmidt M, Klein H-U, Naj AC, Hamilton-Nelson KL, Larson EB (2021). Novel Alzheimer disease risk loci and pathways in African American individuals using the African genome resources panel: a meta-analysis. JAMA Neurol.

[7] Blue EE, Arvr H, Mukherjee S, Wijsman EM, Thornton TA (2019). Local ancestry at APOE modifies Alzheimer’s disease risk in Caribbean Hispanics. Alzheimers Dement.

[8] Graham SE, Clarke SL, Wu K-HH, Kanoni S, Zajac GJM, Ramdas S (2021). The power of genetic diversity in genome-wide association studies of lipids. Nature..

[9] Mahajan A, Spracklen CN, Zhang W, Ng MCY, Petty LE, Kitajima H (2022). Multi-ancestry genetic study of type 2 diabetes highlights the power of diverse populations for discovery and translation. Nat Genet.

[10] McCartney DL, Min JL, Richmond RC, Lu AT, Sobczyk MK, Davies G (2021). Genome-wide association studies identify 137 genetic loci for DNA methylation biomarkers of aging. Genome Biol.

[11] Laisk T, Soares ALG, Ferreira T, Painter JN, Censin JC, Laber S (2020). The genetic architecture of sporadic and multiple consecutive miscarriage. Nat Commun.

[12] Chen M-H, Raffield LM, Mousas A, Sakaue S, Huffman JE, Moscati A (2020). Trans-ethnic and ancestry-specific blood-cell genetics in 746,667 individuals from 5 global populations. Cell..

[13] Shu X, Long J, Cai Q, Kweon S-S, Choi J-Y, Kubo M (2020). Identification of novel breast cancer susceptibility loci in meta-analyses conducted among Asian and European descendants. Nat Commun.

[14] Tin A, Marten J, Halperin Kuhns VL, Li Y, Wuttke M, Kirsten H (2019). Target genes, variants, tissues and transcriptional pathways influencing human serum urate levels. Nat Genet.

[15] Teumer A, Li Y, Ghasemi S, Prins BP, Wuttke M, Hermle T (2019). Genome-wide association meta-analyses and fine-mapping elucidate pathways influencing albuminuria. Nat Commun.

[16] Wuttke M, Li Y, Li M, Sieber KB, Feitosa MF, Gorski M (2019). A catalog of genetic loci associated with kidney function from analyses of a million individuals. Nat Genet.

[17] Morris AP, Le TH, Wu H, Akbarov A, van der Most PJ, Hemani G (2019). Trans-ethnic kidney function association study reveals putative causal genes and effects on kidney-specific disease aetiologies. Nat Commun.

[18] Daya M, Rafaels N, Brunetti TM, Chavan S, Levin AM, Shetty A (2019). Association study in African-admixed populations across the Americas recapitulates asthma risk loci in non-African populations. Nat Commun.

[19] Shigemizu D, Mitsumori R, Akiyama S, Miyashita A, Morizono T, Higaki S (2021). Ethnic and trans-ethnic genome-wide association studies identify new loci influencing Japanese Alzheimer’s disease risk. Transl Psychiatry..

[20] Kang S, Gim J, Lee J, Gunasekaran TI, Choi KY, Lee JJ (2021). Potential novel genes for late-onset Alzheimer’s disease in East-Asian descent identified by APOE-stratified genome-wide association study. J Alzheimers Dis.

[21] Conti DV, Darst BF, Moss LC, Saunders EJ, Sheng X, Chou A (2021). Trans-ancestry genome-wide association meta-analysis of prostate cancer identifies new susceptibility loci and informs genetic risk prediction. Nat Genet.

[22] Ntalla I, Weng L-C, Cartwright JH, Hall AW, Sveinbjornsson G, Tucker NR (2020). Multi-ancestry GWAS of the electrocardiographic PR interval identifies 202 loci underlying cardiac conduction. Nat Commun.

[23] Bentley AR, Sung YJ, Brown MR, Winkler TW, Kraja AT, Ntalla I (2019). Multi-ancestry genome-wide gene-smoking interaction study of 387,272 individuals identifies new loci associated with serum lipids. Nat Genet.

[24] Chang CC, Chow CC, Tellier LC, Vattikuti S, Purcell SM, Lee JJ (2015). Second-generation PLINK: rising to the challenge of larger and richer datasets. Gigascience..

[25] Mägi R, Horikoshi M, Sofer T, Mahajan A, Kitajima H, Franceschini N (2017). Trans-ethnic meta-regression of genome-wide association studies accounting for ancestry increases power for discovery and improves fine-mapping resolution. Hum Mol Genet.

[26] Ochoa D, Hercules A, Carmona M, Suveges D, Gonzalez-Uriarte A, Malangone C (2021). Open Targets Platform: supporting systematic drug-target identification and prioritisation. Nucleic Acids Res.

[27] Zeng B, Bendl J, Kosoy R, Fullard JF, Hoffman GE, Roussos P (2022). Multi-ancestry eQTL meta-analysis of human brain identifies candidate causal variants for brain-related traits. Nat Genet.

[28] Steele NZR, Carr JS, Bonham LW, Geier EG, Damotte V, Miller ZA (2017). Fine-mapping of the human leukocyte antigen locus as a risk factor for Alzheimer disease: a case–control study. PLoS Med.

[29] Hirata J, Hosomichi K, Sakaue S, Kanai M, Nakaoka H, Ishigaki K (2019). Genetic and phenotypic landscape of the major histocompatibilty complex region in the Japanese population. Nat Genet..

[30] Sánchez-Juan P, Moreno S, de Rojas I, Hernández I, Valero S, Alegret M (2019). The MAPT H1 haplotype is a risk factor for Alzheimer’s disease in APOE ε4 non-carriers. Front Aging Neurosci.

[31] Wightman DP, Jansen IE, Savage JE, Shadrin AA, Bahrami S, Holland D (2021). A genome-wide association study with 1,126,563 individuals identifies new risk loci for Alzheimer’s disease. Nat Genet.

[32] Schwartzentruber J, Cooper S, Liu JZ, Barrio-Hernandez I, Bello E, Kumasaka N (2021). Genome-wide meta-analysis, fine-mapping and integrative prioritization implicate new Alzheimer’s disease risk genes. Nat Genet.

[33] Sirkis DW, Geier EG, Bonham LW, Karch CM, Yokoyama JS (2019). Recent advances in the genetics of frontotemporal dementia. Curr Genet Med Rep.

[34] Nalls MA, Blauwendraat C, Vallerga CL, Heilbron K, Bandres-Ciga S, Chang D (2019). Identification of novel risk loci, causal insights, and heritable risk for Parkinson’s disease: a meta-genome wide association study. Lancet Neurol.

[35] Nalls MA, Blauwendraat C, Sargent L, Vitale D, Leonard H, Iwaki H (2021). Evidence for GRN connecting multiple neurodegenerative diseases. Brain Commun.

[36] Miyashita A, Koike A, Jun G, Wang L-S, Takahashi S, Matsubara E (2013). SORL1 is genetically associated with late-onset Alzheimer’s disease in Japanese, Koreans and Caucasians. PLoS ONE.

[37] Rajabli F, Feliciano BE, Celis K, Hamilton-Nelson KL, Whitehead PL, Adams LD (2018). Ancestral origin of ApoE ε4 Alzheimer disease risk in Puerto Rican and African American populations. PLoS Genet.

[38] Tosto G, Fu H, Vardarajan BN, Lee JH, Cheng R, Reyes-Dumeyer D (2015). F-box/LRR-repeat protein 7 is genetically associated with Alzheimer’s disease. Ann Clin Transl Neurol.

[39] Chen DT, Jiang X, Akula N, Shugart YY, Wendland JR, Steele CJM (2011). Genome-wide association study meta-analysis of European and Asian-ancestry samples identifies three novel loci associated with bipolar disorder. Mol Psychiatry.

[40] Kent WJ, Sugnet CW, Furey TS, Roskin KM, Pringle TH, Zahler AM (2002). The human genome browser at UCSC. Genome Res.

[41] Schizophrenia Working Group of the Psychiatric Genomics Consortium, Ripke S, Neale BM, Corvin A, Walters JTR, Farh K-H, et al. Biological insights from 108 Schizophrenia-Associated Genetic Loci. Nature. 2014;511:421. 10.1038/nature13595 PMC4112379 25056061

[42] Lam M, Chen C-Y, Li Z, Martin AR, Bryois J, Ma X (2019). Comparative genetic architectures of schizophrenia in East Asian and European populations. Nat Genet.

[43] Ikeda M, Takahashi A, Kamatani Y, Momozawa Y, Saito T, Kondo K (2019). Genome-wide association study detected novel susceptibility genes for schizophrenia and shared trans-populations/diseases genetic effect. Schizophr Bull.

[44] Goes FS, Hamshere ML, Seifuddin F, Pirooznia M, Belmonte-Mahon P, Breuer R (2012). Genome-wide association of mood-incongruent psychotic bipolar disorder. Transl Psychiatry.

[45] Ruderfer DM, Fanous AH, Ripke S, McQuillin A, Amdur RL (2014). Schizophrenia Working Group of the Psychiatric Genomics Consortium, et al. Polygenic dissection of diagnosis and clinical dimensions of bipolar disorder and schizophrenia. Mol Psychiatry.

[46] Mullins N, Forstner AJ, O’Connell KS, Coombes B, Coleman JRI, Qiao Z (2021). Genome-wide association study of more than 40,000 bipolar disorder cases provides new insights into the underlying biology. Nat Genet.

[47] Drange OK, Smeland OB, Shadrin AA, Finseth PI, Witoelar A, Frei O (2019). Genetic overlap between Alzheimer’s disease and bipolar disorder implicates the MARK2 and VAC14 Genes. Front Neurosci.

[48] Li W, Cai X, Li H-J, Song M, Zhang C-Y, Yang Y (2021). Independent replications and integrative analyses confirm TRANK1 as a susceptibility gene for bipolar disorder. Neuropsychopharmacology..

[49] Jiang X, Detera-Wadleigh SD, Akula N, Mallon BS, Hou L, Xiao T (2019). Sodium valproate rescues expression of TRANK1 in iPSC-derived neural cells that carry a genetic variant associated with serious mental illness. Mol Psychiatry.

[50] Gambuzza ME, Sofo V, Salmeri FM, Soraci L, Marino S, Bramanti P (2014). Toll-like receptors in Alzheimer’s disease: a therapeutic perspective. CNS Neurol Disord Drug Targets.

[51] Wolters FJ, Boender J, de Vries PS, Sonneveld MA, Koudstaal PJ, de Maat MP (2018). Von Willebrand factor and ADAMTS13 activity in relation to risk of dementia: a population-based study. Sci Rep.

[52] Li QS, De, Muynck L (2021). Differentially expressed genes in Alzheimer’s disease highlighting the roles of microglia genes including OLR1 and astrocyte gene CDK2AP1. Brain Behav Immun Health.

[53] Faux NG, Rembach A, Wiley J, Ellis KA, Ames D, Fowler CJ (2014). An anemia of Alzheimer’s disease. Mol Psychiatry.

[54] Ramos-Cejudo J, Wisniewski T, Marmar C, Zetterberg H, Blennow K, de Leon MJ (2018). Traumatic Brain Injury and Alzheimer’s Disease: the cerebrovascular link. EBioMedicine..

[55] Dean M. The human ATP-binding cassette (ABC) transporter superfamily. USA: National Center for Biotechnology Information; 2002.

[56] Cao QT, Aguiar JA, Tremblay BJ-M, Abbas N, Tiessen N, Revill S (2020). ABCF1 regulates dsDNA-induced immune responses in human airway epithelial cells. Front Cell Infect Microbiol.

[57] Momtazmanesh S, Perry G, Rezaei N (2020). Toll-like receptors in Alzheimer’s disease. J Neuroimmunol.

[58] Courtney SC, Di H, Stockman BM, Liu H, Scherbik SV, Brinton MA (2012). Identification of novel host cell binding partners of Oas1b, the protein conferring resistance to flavivirus-induced disease in mice. J Virol.

[59] Vittor AY, Long M, Chakrabarty P, Aycock L, Kollu V, DeKosky ST (2020). West nile virus-induced neurologic sequelae-relationship to neurodegenerative cascades and dementias. Curr Trop Med Rep.

[60] de Oliveira J, Kucharska E, Garcez ML, Rodrigues MS, Quevedo J, Moreno-Gonzalez I (2021). Inflammatory cascade in Alzheimer’s disease pathogenesis: a review of experimental findings. Cells..

[61] Skol AD, Scott LJ, Abecasis GR, Boehnke M (2006). Joint analysis is more efficient than replication-based analysis for two-stage genome-wide association studies. Nat Genet.

[62] Vardarajan BN, Reyes-Dumeyer D, Piriz AL, Lantigua RA, Medrano M, Rivera D, et al. Progranulin mutations in clinical and neuropathological Alzheimer’s disease. Alzheimers Dement. 2022. 10.1002/alz.12567. 10.1002/alz.12567 PMC9360185 35258170

[63] Tönjes A, Scholz M, Krüger J, Krause K, Schleinitz D, Kirsten H (2018). Genome-wide meta-analysis identifies novel determinants of circulating serum progranulin. Hum Mol Genet.

[64] Liu C, Yu J (2019). Genome-wide association studies for cerebrospinal fluid soluble TREM2 in Alzheimer’s disease. Front Aging Neurosci.

[65] Hou XH, Bi YL, Tan MS, Xu W, Li JQ, Shen XN (2019). Genome-wide association study identifies Alzheimer’s risk variant in MS4A6A influencing cerebrospinal fluid sTREM2 levels. Neurobiol Aging.

[66] Deming Y, Filipello F, Cignarella F, Cantoni C, Hsu S, Mikesell R (2019). The MS4A gene cluster is a key modulator of soluble TREM2 and Alzheimer’s disease risk. Sci Transl Med.

[67] Acosta-Uribe J, Aguillón D, Cochran JN, Giraldo M, Madrigal L, Killingsworth BW (2022). A neurodegenerative disease landscape of rare mutations in Colombia due to founder effects. Genome Med.

[68] Jiao B, Liu X, Tang B, Hou L, Zhou L, Zhang F (2014). Investigation of TREM2, PLD3, and UNC5C variants in patients with Alzheimer’s disease from mainland China. Neurobiol Aging.

[69] Reitz C, Jun G, Naj A, Rajbhandary R, Vardarajan BN, Wang L-S (2013). Variants in the ATP-binding cassette transporter (ABCA7), Apolipoprotein E ϵ4, and the risk of late-onset Alzheimer disease in African Americans. JAMA..

[70] Gianattasio KZ, Prather C, Glymour MM, Ciarleglio A, Power MC (2019). Racial disparities and temporal trends in dementia misdiagnosis risk in the United States. Alzheimers Dement.

[71] Beach TG, Monsell SE, Phillips LE, Kukull W (2012). Accuracy of the clinical diagnosis of Alzheimer disease at National Institute on Aging Alzheimer Disease Centers, 2005–10. J Neuropathol Exp Neurol.

[72] Jansen IE, Savage JE, Watanabe K, Bryois J, Williams DM, Steinberg S (2019). Genome-wide meta-analysis identifies new loci and functional pathways influencing Alzheimer’s disease risk. Nat Genet.

[73] Jonsson T, Atwal JK, Steinberg S, Snaedal J, Jonsson PV, Bjornsson S (2012). A mutation in APP protects against Alzheimer’s disease and age-related cognitive decline. Nature..

[74] Sirkis DW, Bonham LW, Johnson TP, La Joie R, Yokoyama JS (2022). Dissecting the clinical heterogeneity of early-onset Alzheimer’s disease. Mol Psychiatry.

[75] Deeks JJ, Higgins JPT, Altman DG, on behalf of the Cochrane Statistical Methods Group. Analysing data and undertaking meta‐analyses. Cochrane Handbook for Systematic Reviews of Interventions. 2019:241–84.

[76] Gay NR, Gloudemans M, Antonio ML, Abell NS, Balliu B, Park Y (2020). Impact of admixture and ancestry on eQTL analysis and GWAS colocalization in GTEx. Genome Biol.

[77] Zhu Z, Zhang F, Hu H, Bakshi A, Robinson MR, Powell JE (2016). Integration of summary data from GWAS and eQTL studies predicts complex trait gene targets. Nat Genet.

[78] King EA, Wade Davis J, Degner JF (2019). Are drug targets with genetic support twice as likely to be approved? Revised estimates of the impact of genetic support for drug mechanisms on the probability of drug approval. PLoS Genet.

[79] Liu B, Gloudemans M, Rao AS, Ingelsson E, Montgomery SB (2019). Abundant associations with gene expression complicate GWAS follow-up. Nat Genet.

[80] Murphy AE, Schilder BM, Skene NG (2021). MungeSumstats: a bioconductor package for the standardization and quality control of many GWAS summary statistics. Bioinformatics..

[81] Abraham G, Inouye M (2014). Fast principal component analysis of large-scale genome-wide data. PLoS ONE.

[82] Montinaro F, Busby GBJ, Pascali VL, Myers S, Hellenthal G, Capelli C (2015). Unravelling the hidden ancestry of American admixed populations. Nat Commun.

[83] Tokunaga K, Ohashi J, Bannai M, Juji T. Genetic link between Asians and native Americans: evidence from HLA genes and haplotypes. Hum Immunol. 2001;62:1001–8. 10.1016/s0198-8859(01)00301-9 11543902

