# Supplementary material for: Multi-ancestry meta-analysis and fine-mapping in Alzheimer’s disease
Source: Mol Psychiatry. 2023 May 18;28(7):3121–32. doi: 10.1038/s41380-023-02089-w (PMC10615750; doi:10.1038/s41380-023-02089-w)
Supplement: Supplementary file 1 — Supplemental Material [file 41380_2023_2089_MOESM1_ESM.docx]

# SUPPLEMENTARY INFORMATION

Multi-ancestry meta-analysis and fine-mapping in Alzheimer’s Disease

**
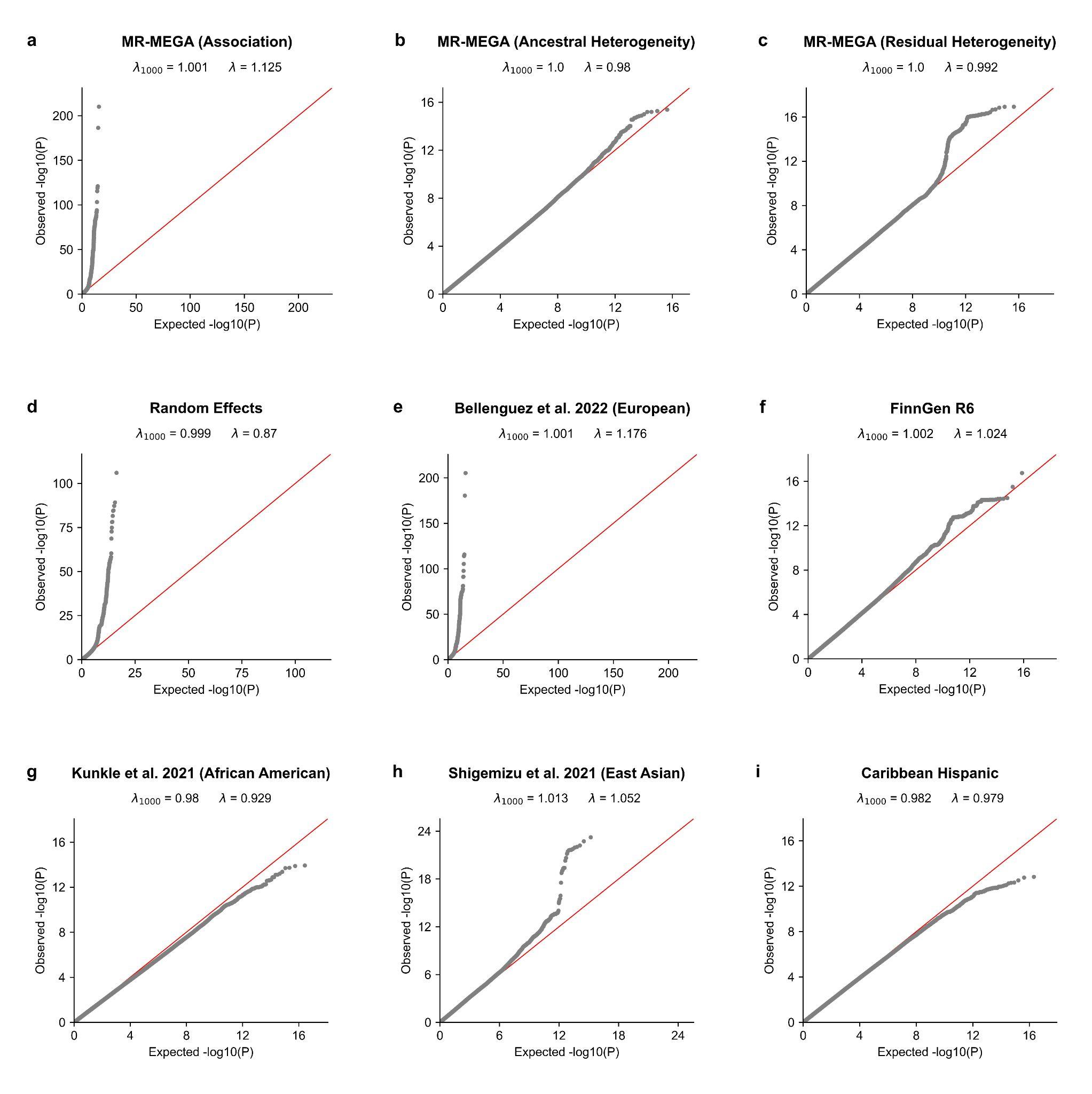
**

**Fig. S1**: Quantile-quantile plots and corresponding genomic inflation estimates, with and without scaling to 1000 cases and 1000 controls. MR-MEGA P-values are shown for association, ancestral heterogeneity, and residual heterogeneity. Chromosome 19 was excluded from all datasets to avoid bias by the *APOE* region. All summary statistics were filtered for MAF > 1%.

**
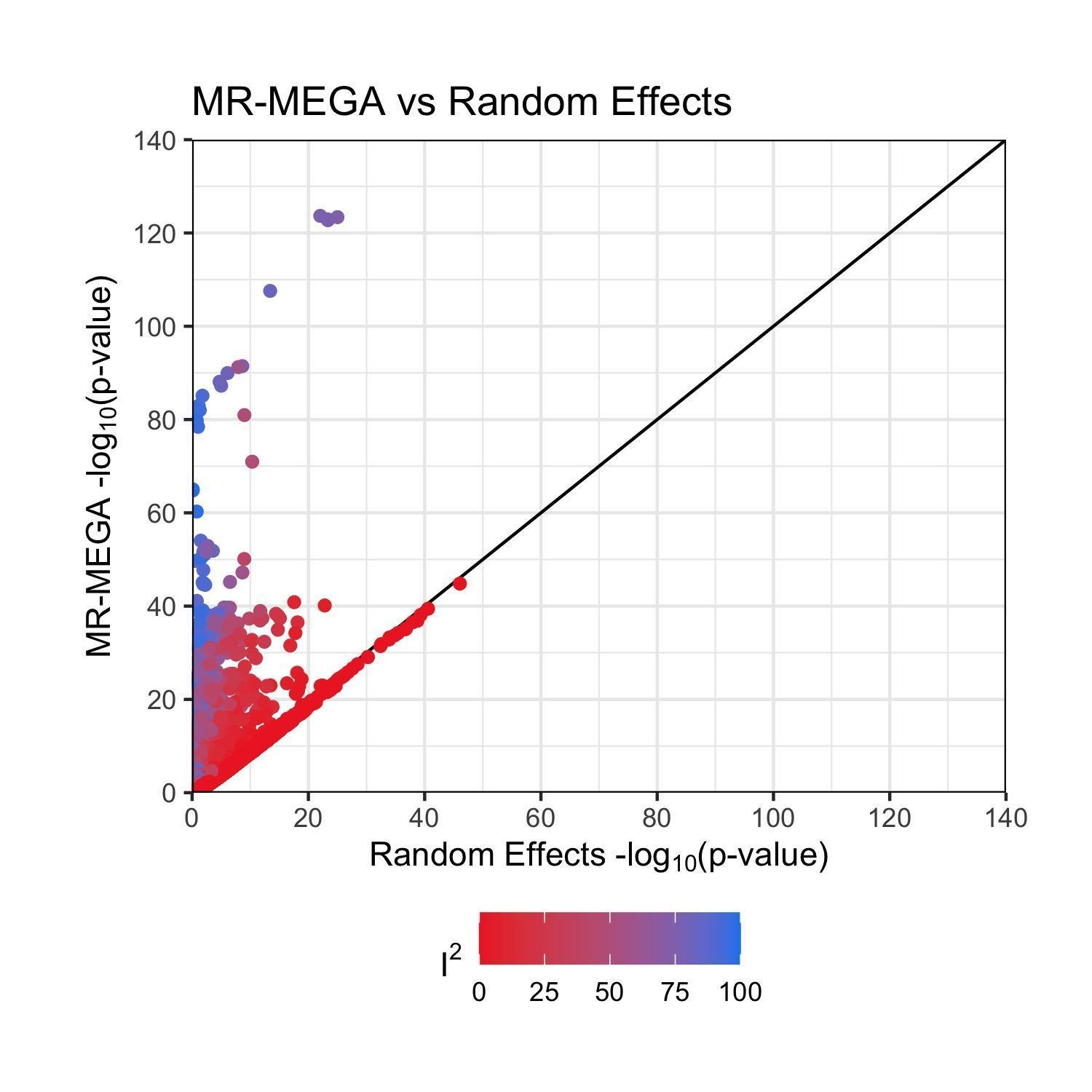
**

**Fig. S2:** MR-MEGA -log_10_ P-values plotted against random effects -log_10_ P-values. SNPs are colored by I^2^ value and are limited to those present in at least 4 datasets.

**
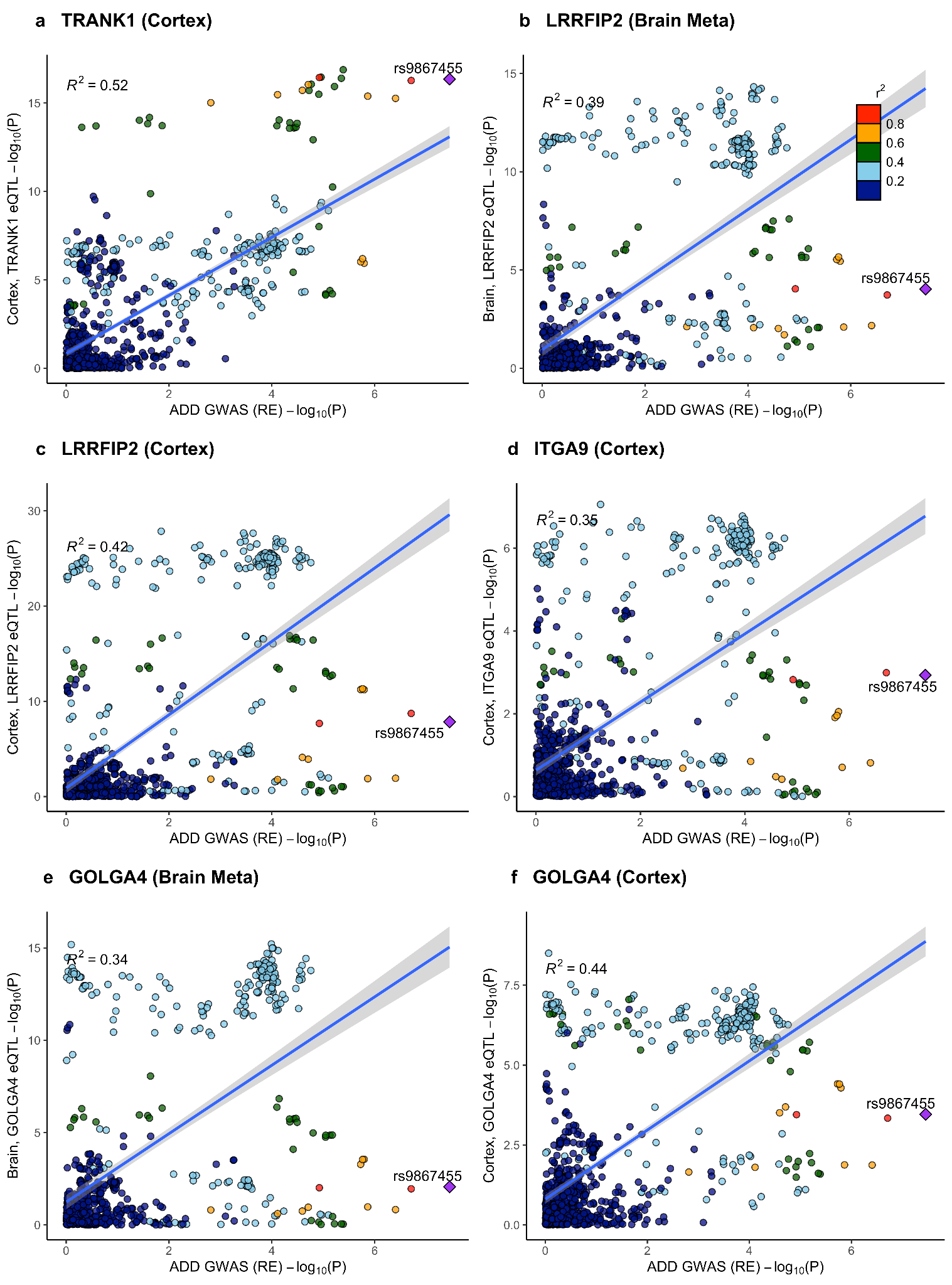
**

**Fig. S3:** LocusCompare plots showing colocalization at the *TRANK1* locus between the random effects meta-analysis results and brain eQTLs (P < 1 x 10^-6^) in genes that were significant in SMR (FDR P < 0.05). Reference LD patterns are based on the European population from 1000 Genomes. Points represent SNPs plotted at their -log_10_ P-values.

**
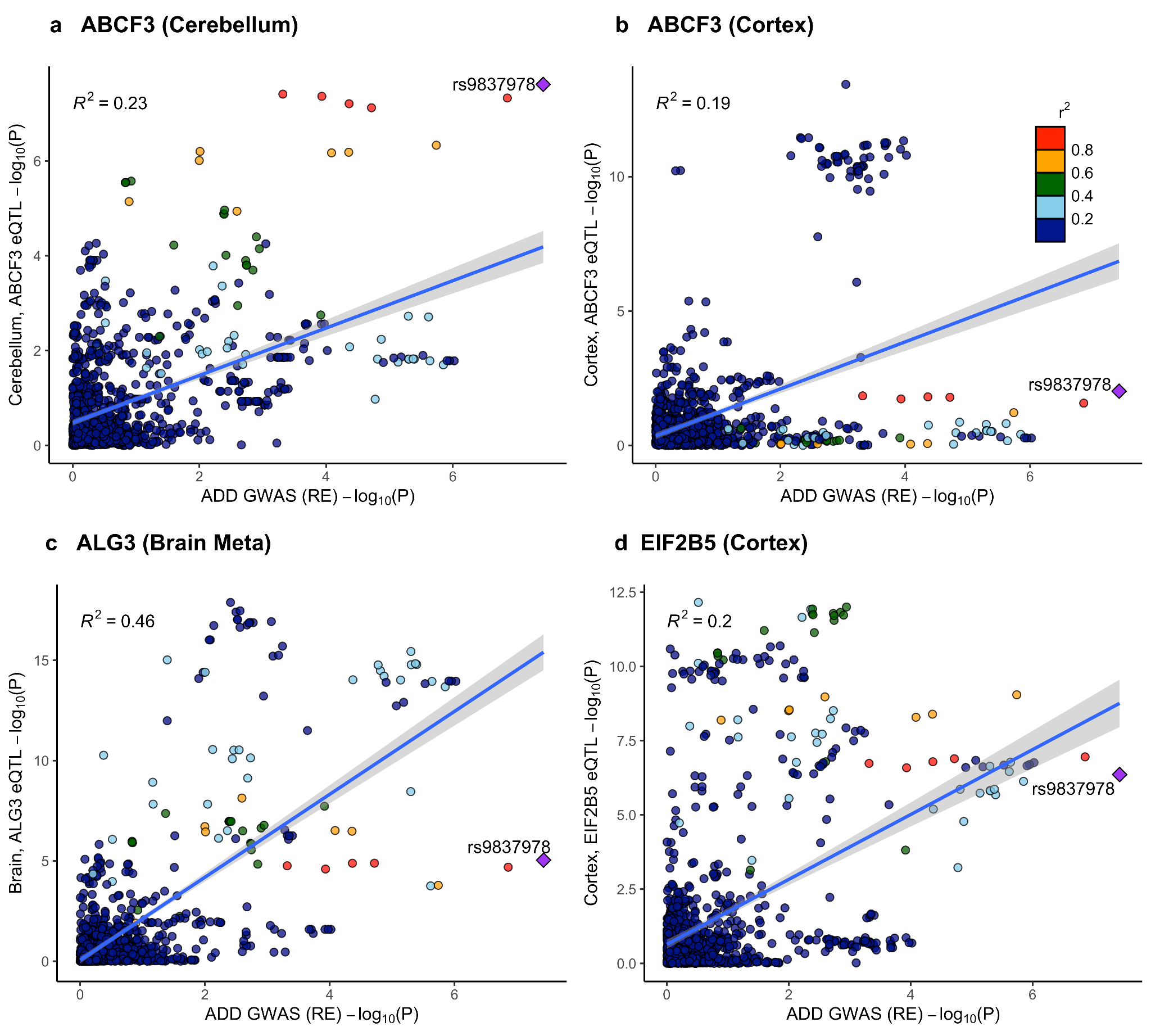
**

**Fig. S4:** LocusCompare plots showing colocalization at the *VWA5B2* locus between the random effects meta-analysis results and brain eQTLs (P < 1 x 10^-6^) in genes that were significant in SMR (FDR P < 0.05). Reference LD patterns are based on the European population from 1000 Genomes. Points represent SNPs plotted at their -log_10_ P-values.

**
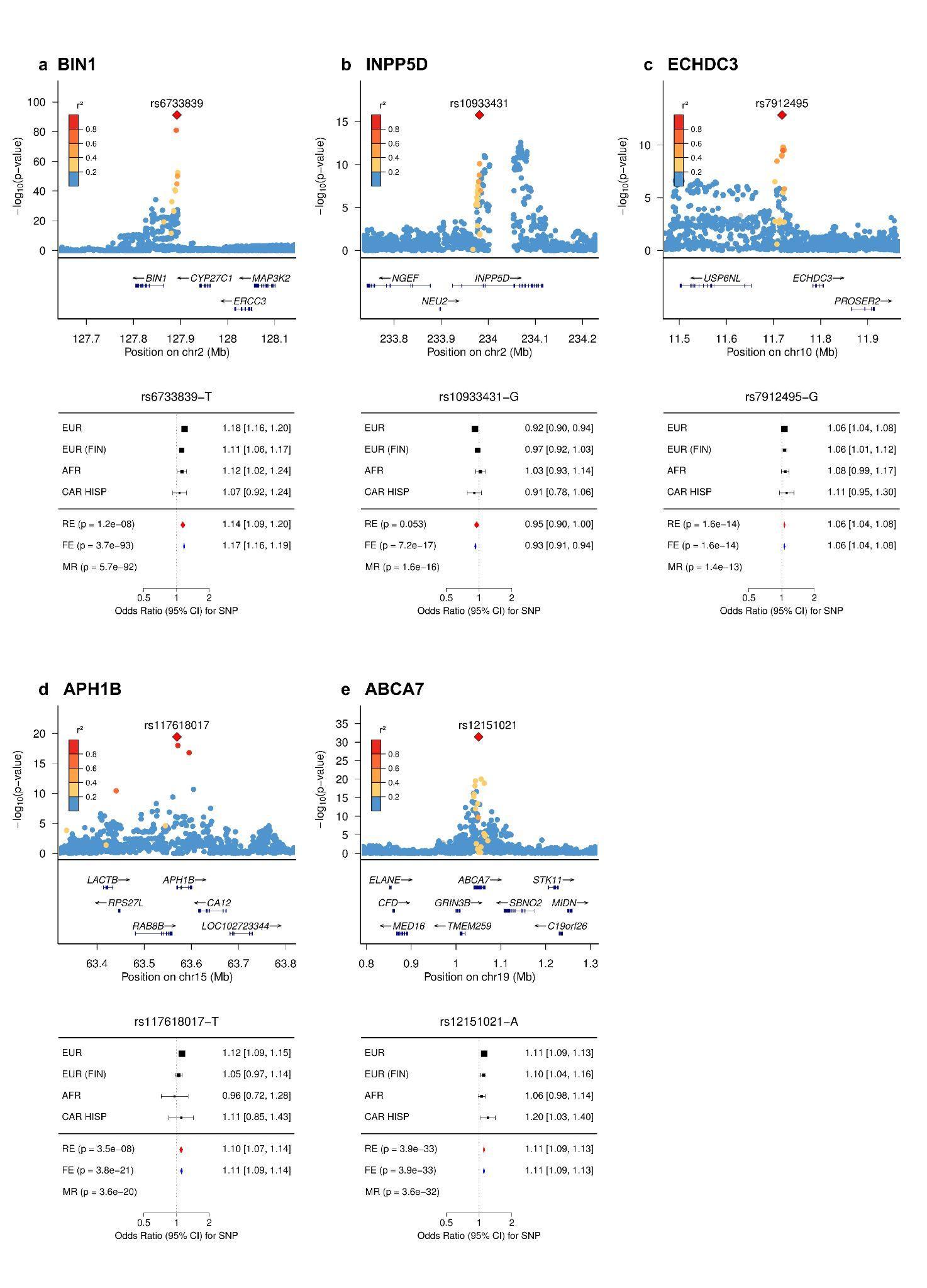
**

**Fig. S5:** LocusZoom and forest plots for SNPs fine-mapped in our study with posterior probability (PP) > 0.8 that have been previously fine-mapped in European studies. Reference LD patterns are based on all populations from 1000 Genomes.


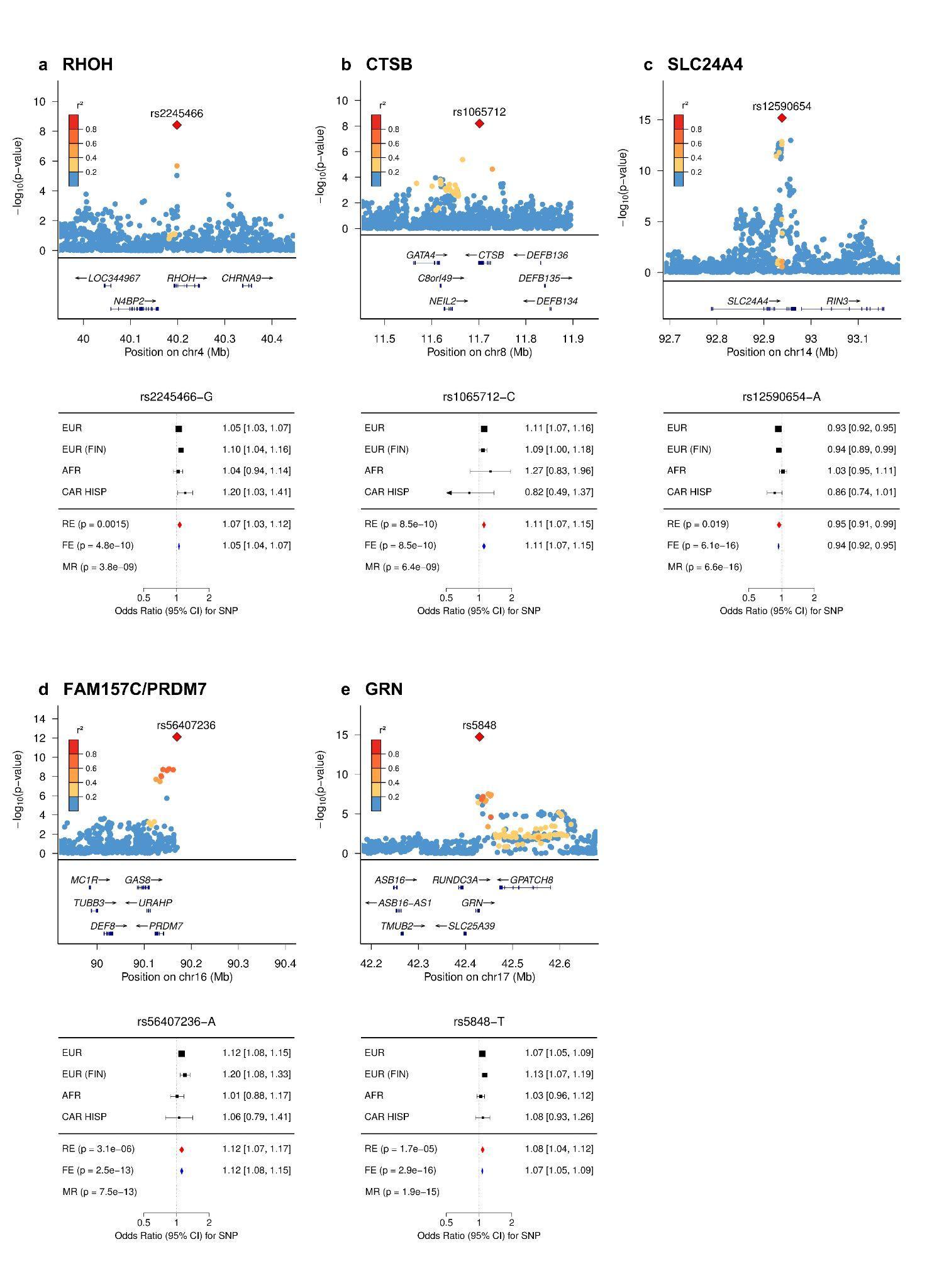


**Fig. S6:** LocusZoom and forest plots for SNPs fine-mapped in our study with posterior probability (PP) > 0.8 that have **not** been previously fine-mapped. Reference LD patterns are based on all populations from 1000 Genomes.

**
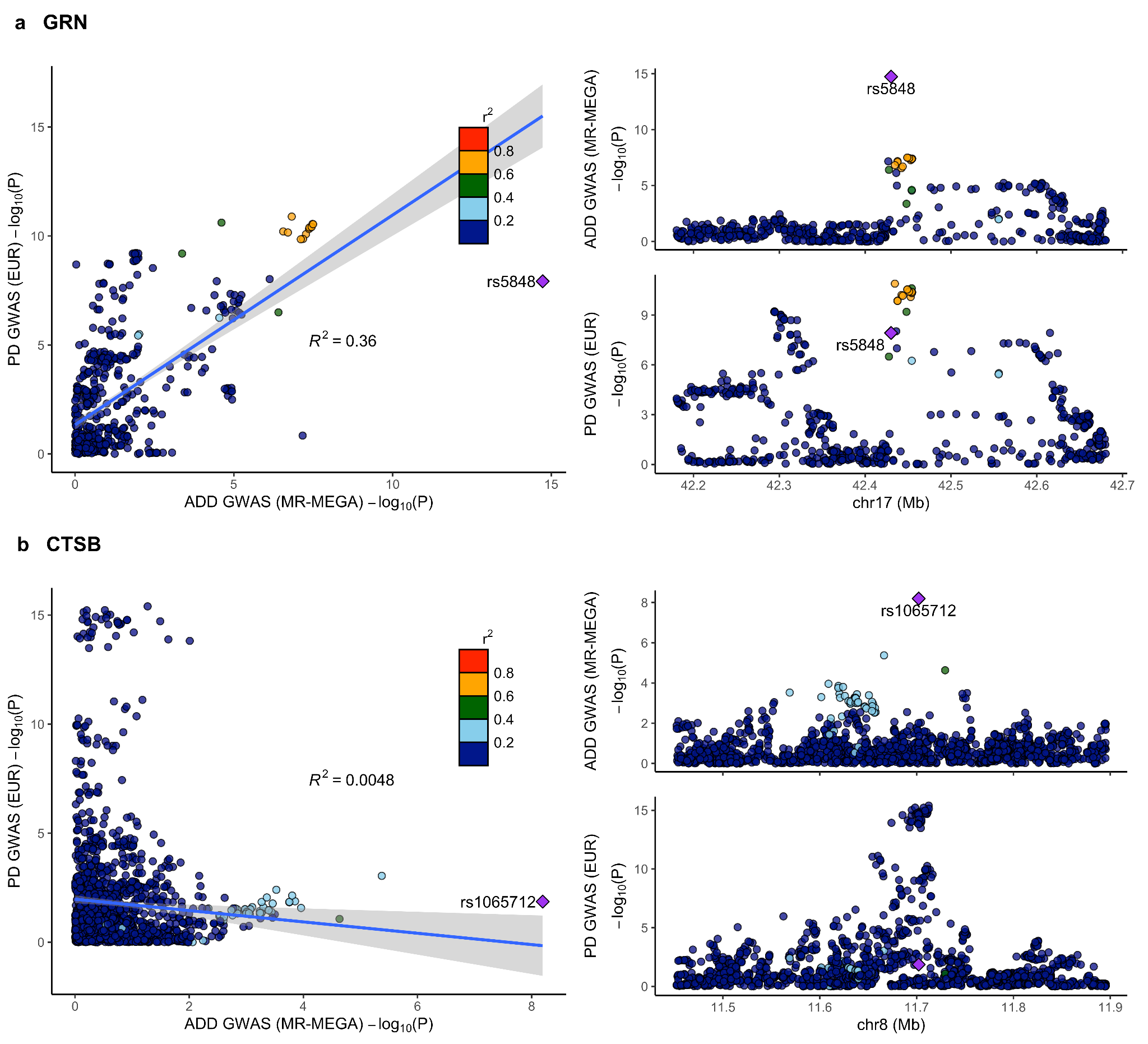
**

**Fig. S7:** LocusCompare plots for a) *GRN* and b) *CTSB* between AD and Parkinson’s disease (PD). Reference LD patterns are based on the European population from 1000 Genomes. Points represent SNPs plotted at their -log_10_ P-values.

**
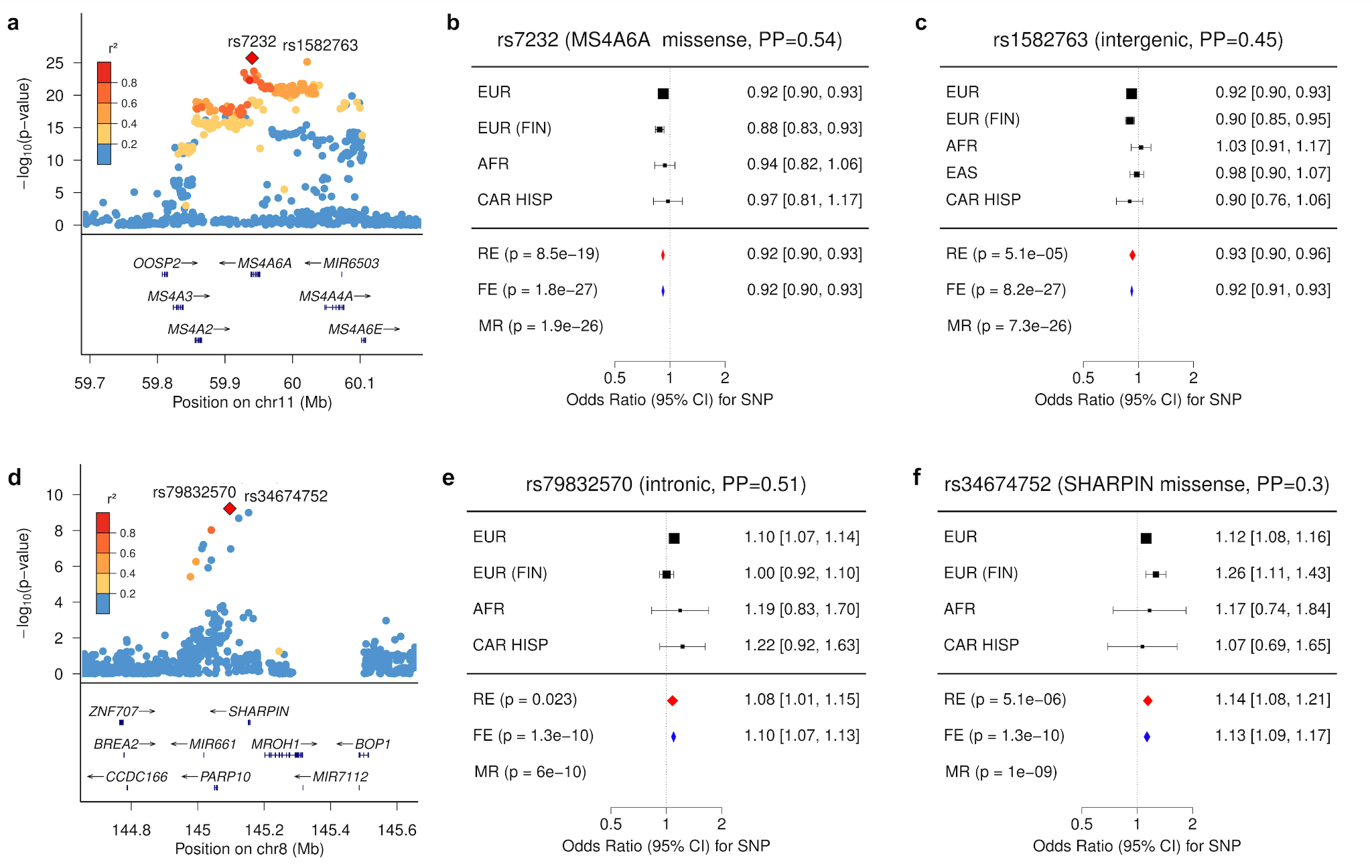
**

**Fig. S8:** LocusZoom and forest plots for loci that were fine-mapped to a missense variant with PP ≥ 0.3. a) LocusZoom plot for *MS4A6A* locus highlighting the top two fine-mapped SNPs, rs7232 and rs1582763 b) forest plot for rs7232 c) forest plot for rs1582763 d) LocusZoom plot for *SHARPIN* locus highlighting the top two fine-mapped SNPs, rs79832570 and rs34674752 e) forest plot for rs79832570 f) forest plot for rs34674752.

**
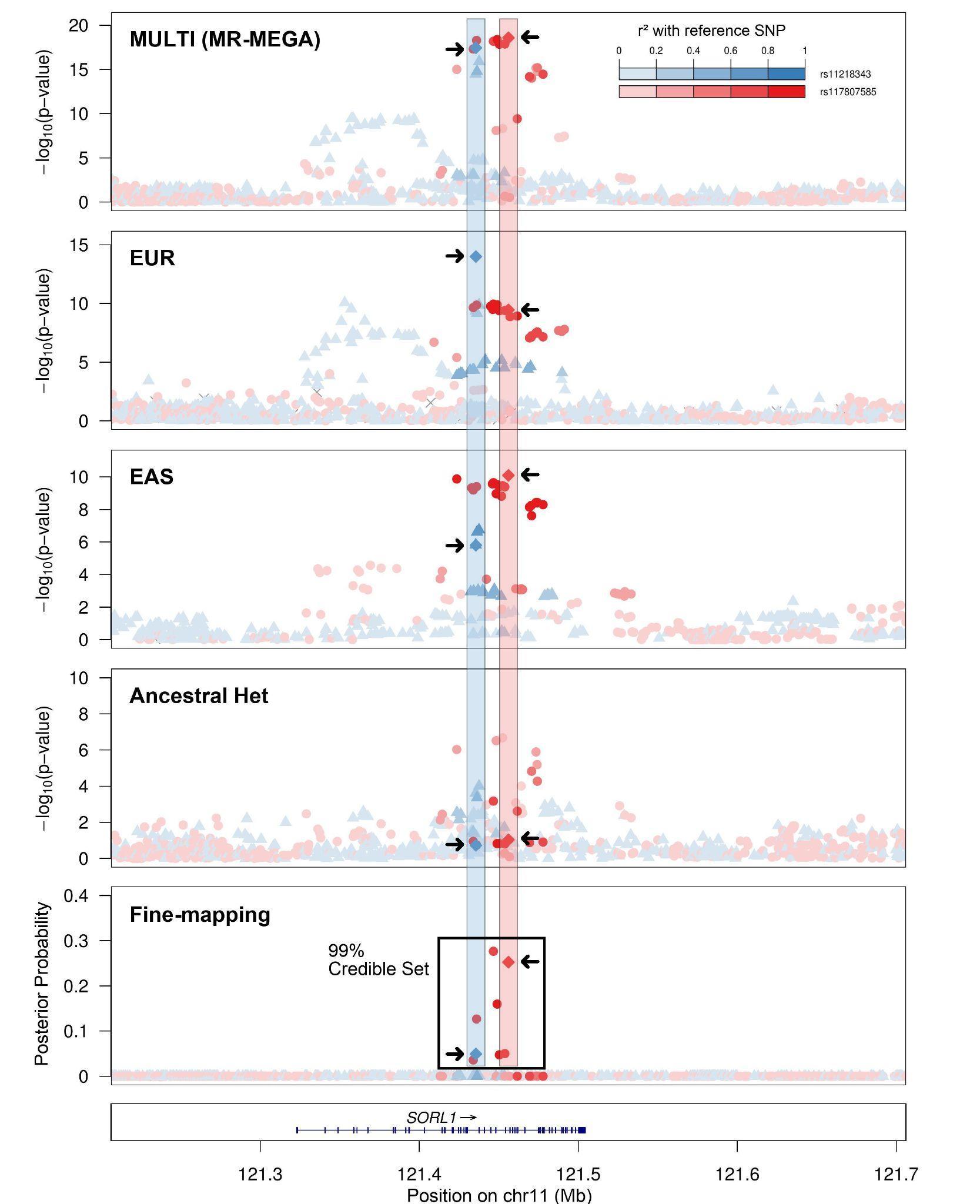
**

**Fig. S9:** LocusZoom plots showing different regional architecture at the *SORL1* locus in East Asian (Shigemizu et al.) versus European (Bellenguez et al.) populations. Diamond points represent the 2 different lead SNPs at this locus, *SORL1*-rs11218343 in Europeans (shown in blue) and *SORL1*-rs117807585 in East Asians (shown in red). Ancestral Het refers to the P-value of heterogeneity that is due to genetic ancestry as defined by MR-MEGA. SNPs are colored by LD with the respective population from 1000 Genomes, or all populations for the multi-ancestry analysis.


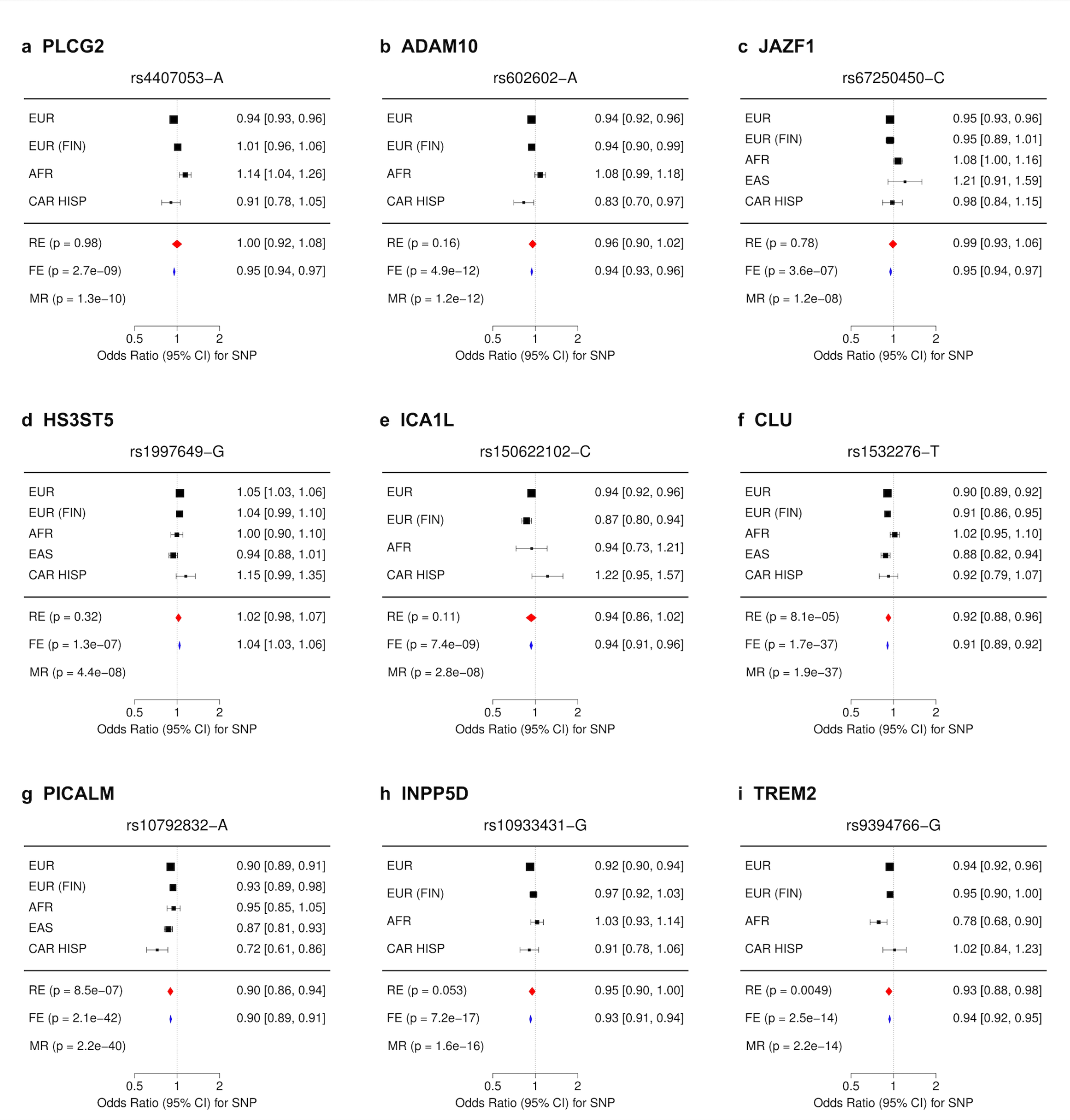

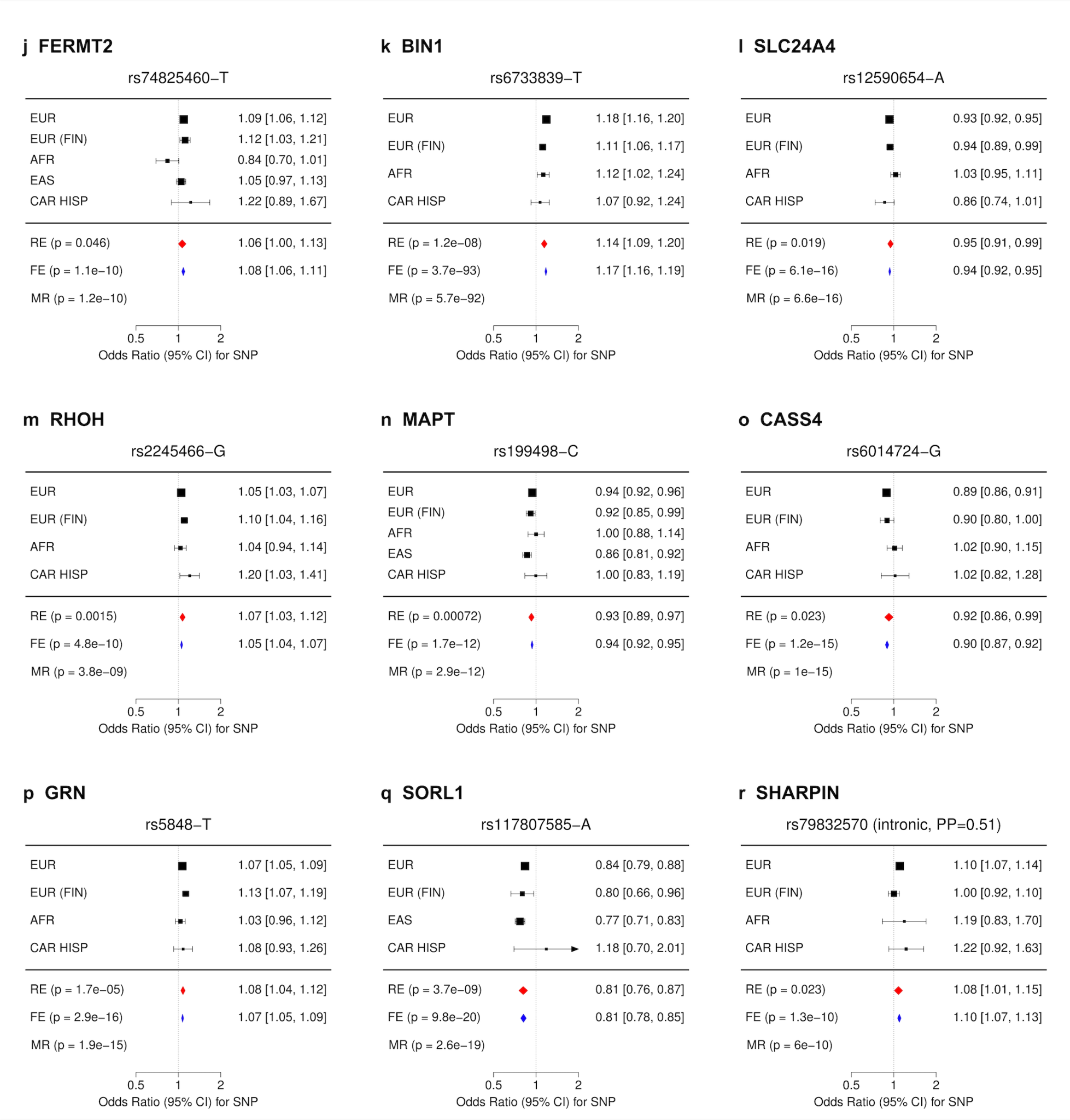


**Fig. S10:** Forest plots for loci with significant heterogeneity (I^2^ > 30%) outside of the *APOE* region.


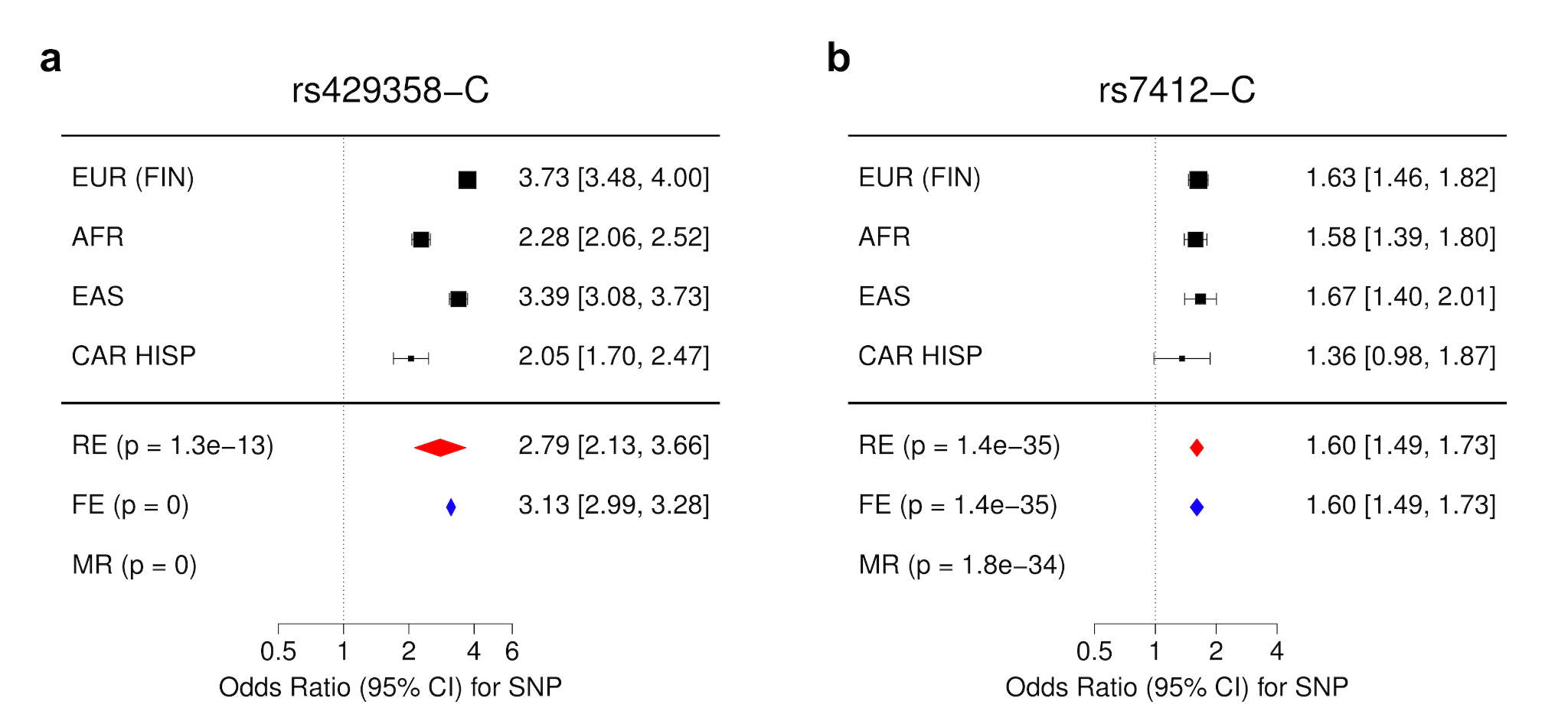


**Fig. S11:** Forest plots for a) *APOE*-rs429358 and b) *APOE*-rs7412.

**
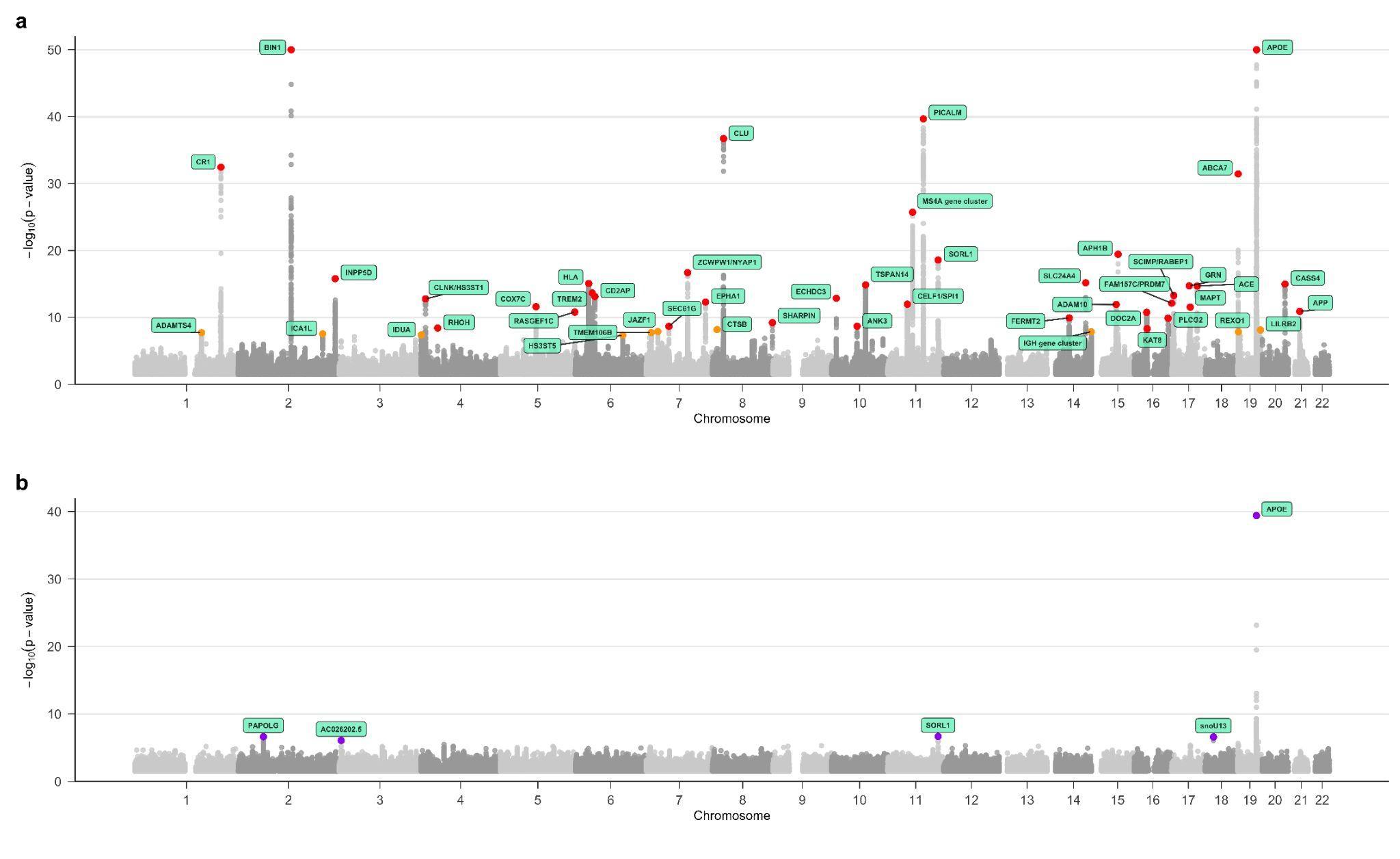
**

**Fig. S12:** a) Manhattan plot for MR-MEGA meta-analysis truncated at -log_10_(P) < 50. Red labeling corresponds to significance at P < 5 x 10^-9^ and orange corresponds to significance at P < 5 x 10^-8^. b) Manhattan plot for P_HET_ from MR-MEGA truncated at -log_10_(P) < 40. Purple labeling corresponds to P < 1 x 10^-6^.

**
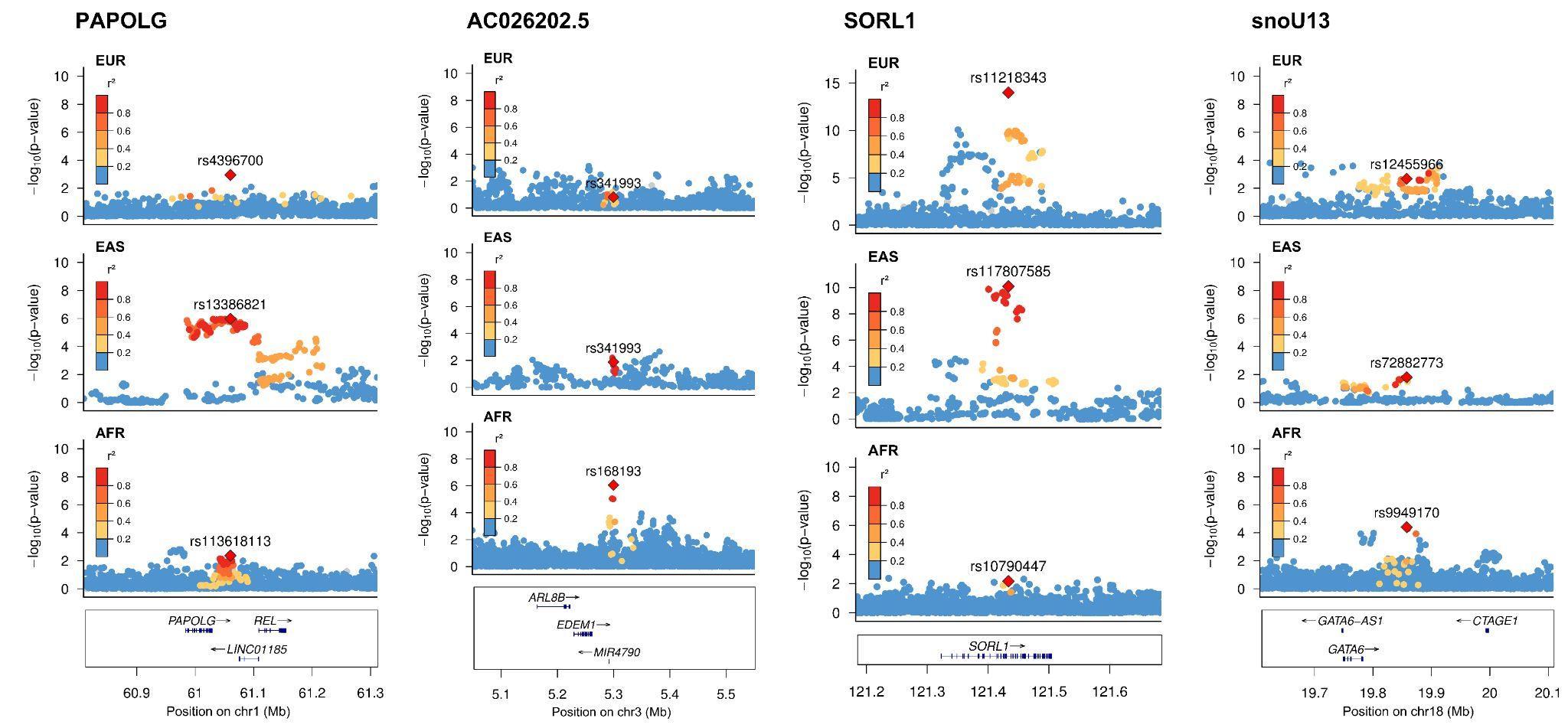
**

**Fig. S13a:** LocusZoom plots of loci showing significant ancestry-related heterogeneity (P_HET_ < 1 x 10^-6^) near *SORL1*, *PAPOLG*, *AC026202.5*, and *snoU13*. Labeled red diamonds correspond to the lead SNP in each ancestry group.


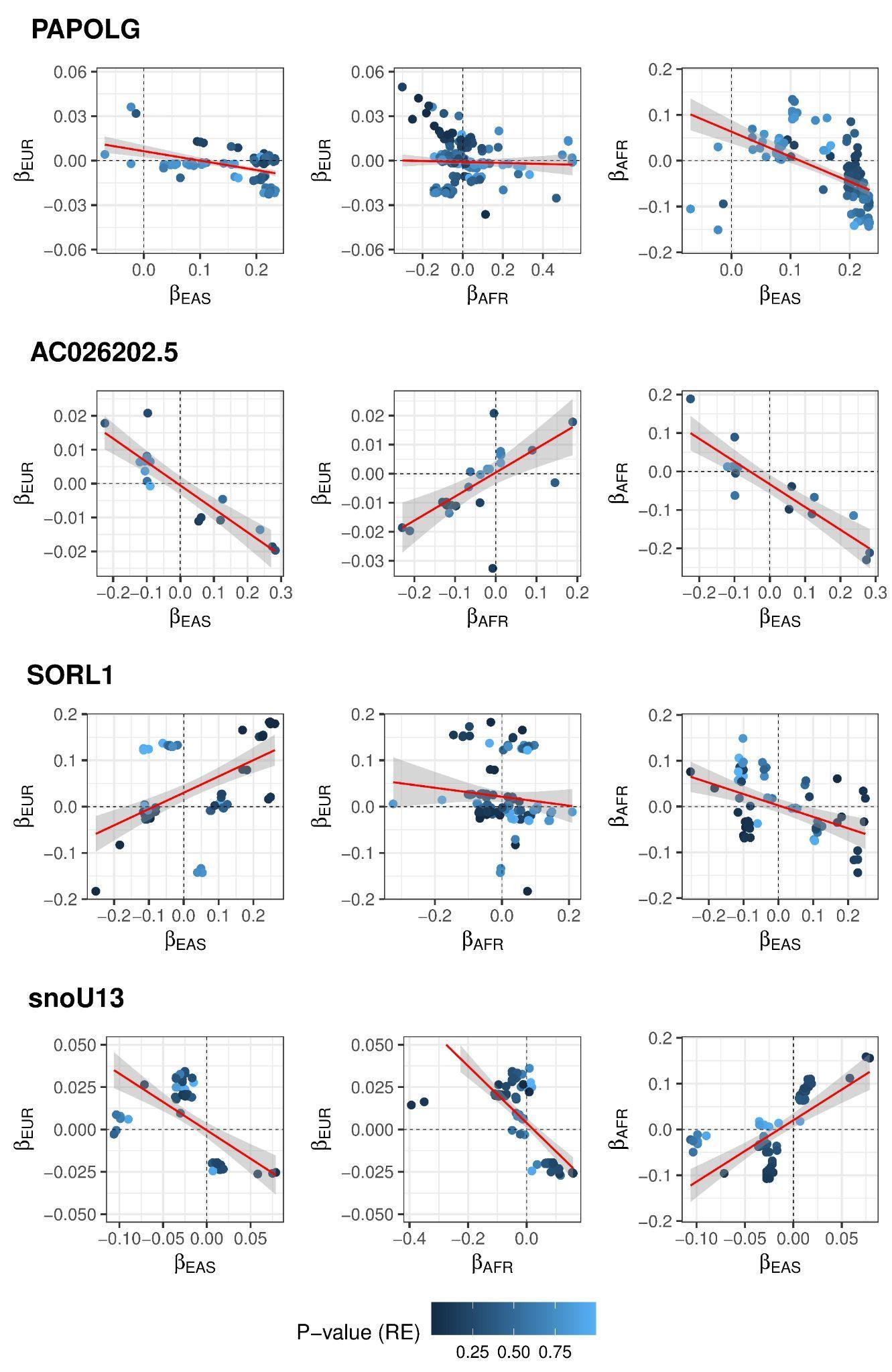


**Fig. S13b:** Beta-beta plots showing effect size correlation of *SORL1*, *PAPOLG*, *AC026202.5*, and *snoU13* across ancestry groups.

**
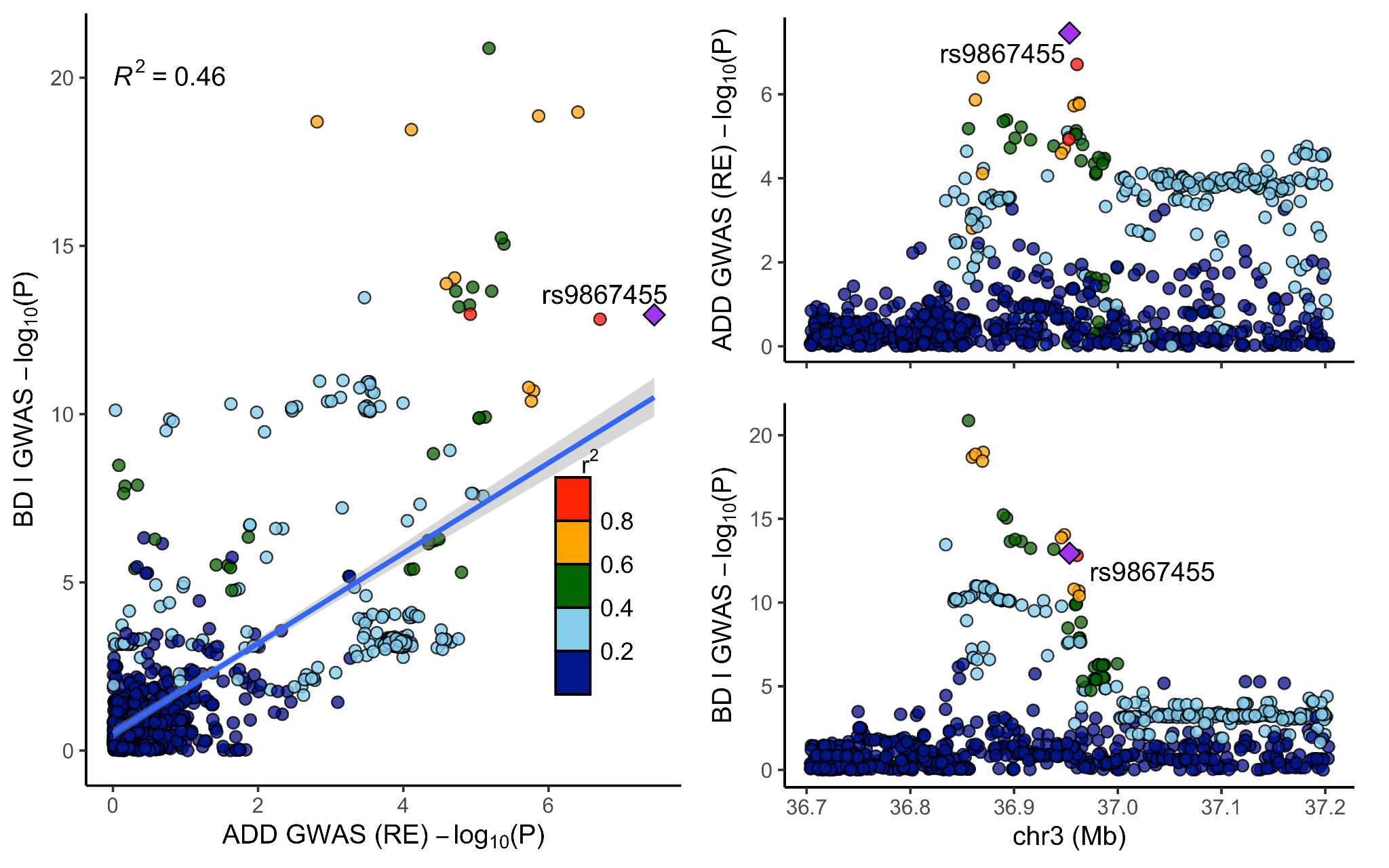
**

**Fig. S14:** LocusCompare plot for bipolar disorder I (BD I) and the random effects ADD meta-analysis at the *TRANK1* locus. Reference LD patterns are based on the European population from 1000 Genomes. Points represent SNPs plotted at their -log_10_ P-values.

**
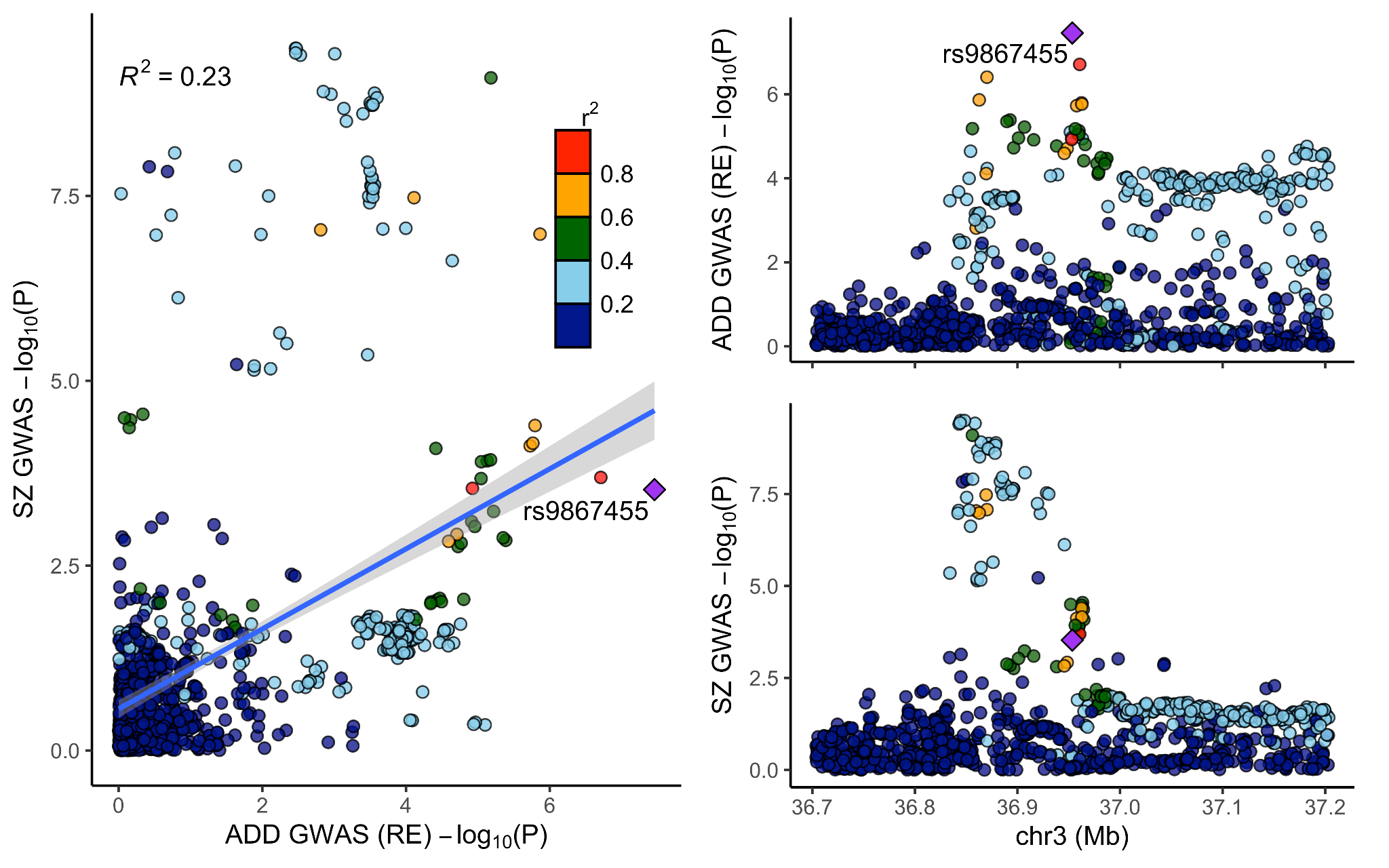
**

**Fig. S15:** LocusCompare plot for schizophrenia (SZ) and the random effects ADD meta-analysis at the *TRANK1* locus. Reference LD patterns are based on the European population from 1000 Genomes. Points represent SNPs plotted at their -log_10_ P-values.


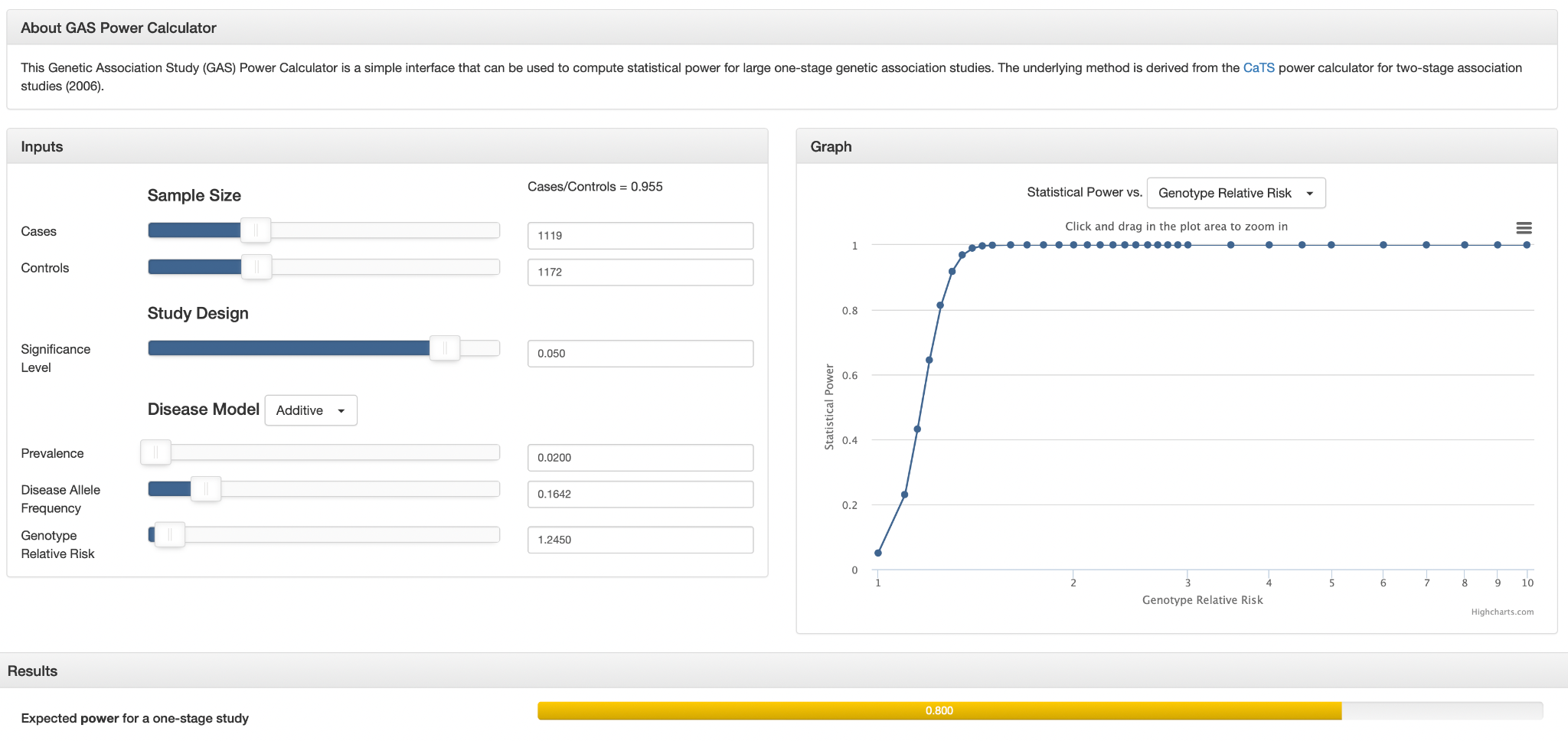


**Fig. S16**: Power calculation for V*WA5B2*-rs9837978 in the GARD cohort using GAS Power Calculator.

**
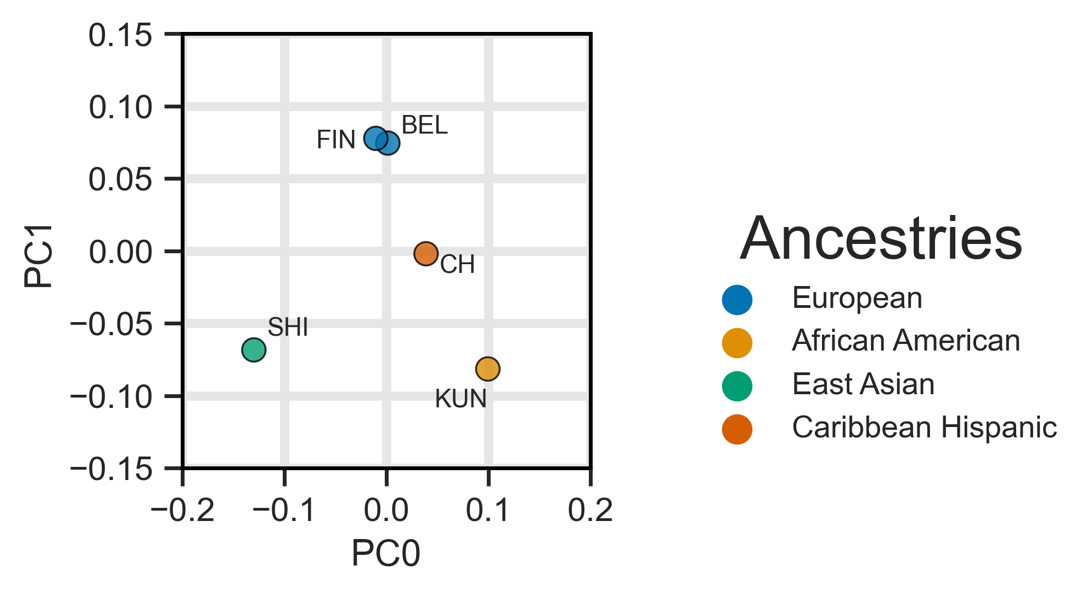
**

**Fig. S17:** The first 2 ancestral principal components (PCs) created and used by MR-MEGA plotted against each other, labeled by dataset (FIN: FinnGen R6, BEL: Bellenguez et al., CH: Caribbean Hispanic, SHI: Shigemizu et al., KUN: Kunkle et al.) and color coded by ancestry group.
